# Centrosome clustering control in osteoclasts through CCR5-mediated signaling

**DOI:** 10.1038/s41598-023-48140-2

**Published:** 2023-11-27

**Authors:** Ji-Won Lee, In-Hee Lee, Haruhisa Watanabe, Yunqing Liu, Kazuaki Sawada, Masashi Maekawa, Shunsuke Uehara, Yasuhiro Kobayashi, Yuuki Imai, Sek Won Kong, Tadahiro Iimura

**Affiliations:** 1 Department of Pharmacology, Faculty and Graduate School of Dental Medicine, Hokkaido University, Sapporo, 060-8586 Japan; 2 Department of Oral Molecular Microbiology, Faculty and Graduate School of Dental Medicine, Hokkaido University, Sapporo, Japan; 3 Computational Health and Informatics Program, Boston Children’s Hospital, Boston, MA USA; 4 NIKON SOLUTIONS CO., LTD., Oi Plant 6-3, Nishioi 1-Chome, Shinagawa-ku, Tokyo Japan; 5 Division of Physiological Chemistry and Metabolism, Graduate School of Pharmaceutical Sciences, Keio University, Tokyo, Japan; 6 Department of Biochemistry, Matsumoto Dental University, Nagano, Japan; 7 Division of Hard Tissue Research, Institute for Oral Science, Matsumoto Dental University, Nagano, Japan; 8 Division of Integrative Pathophysiology, Proteo-Science Center, Ehime University, Ehime, Japan; 9 Department of Pathophysiology, Ehime University Graduate School of Medicine, Ehime, Japan; 10 Department of Pediatrics, Harvard Medical School, Boston, MA USA

**Keywords:** Mechanisms of disease, Chemokines, Bone

## Abstract

Osteoclasts uniquely resorb calcified bone matrices. To exert their function, mature osteoclasts maintain the cellular polarity and directional vesicle trafficking to and from the resorbing bone surface. However, the regulatory mechanisms and pathophysiological relevance of these processes remain largely unexplored. Bone histomorphometric analyses in *Ccr5*-deficient mice showed abnormalities in the morphology and functional phenotype of their osteoclasts, compared to wild type mice. We observed disorganized clustering of nuclei, as well as centrosomes that organize the microtubule network, which was concomitant with impaired cathepsin K secretion in cultured *Ccr5*-deficient osteoclasts. Intriguingly, forced expression of constitutively active Rho or Rac restored these cytoskeletal phenotypes with recovery of cathepsin K secretion. Furthermore, a gene-disease enrichment analysis identified that *PLEKHM1*, a responsible gene for osteopetrosis, which regulates lysosomal trafficking in osteoclasts, was regulated by CCR5. These experimental results highlighted that CCR5-mediated signaling served as an intracellular organizer for centrosome clustering in osteoclasts, which was involved in the pathophysiology of bone metabolism.

## Introduction

Osteoclasts are highly specialized polykaryons that are capable of resorbing calcified bone matrix. Upon differentiation, myeloid precursors of the monocyte/macrophage lineage fuse to each other to form multinuclear osteoclasts^1,2^. To facilitate bone resorption, osteoclasts must undergo a significant cytoskeletal reorganization, involving the rearrangement of filamentous subcellular structures like polarized actin filaments, microtubules, non-polarized intermediate filaments, and septin filaments^3–5^. This reorganization plays a crucial role in various cellular functions, notably in cell adhesion, migration, division and vesicle trafficking. A complex yet organized network of microtubules sustains a polarized structure of osteoclasts that enables them firmly attached to the bone surface through organizing their podosomes, a process that is essential for them to exert their functions, including migration, locomotion, and bone resorption^6–8^.

In animal cells, the centrosome serves as a microtubule-organizing center (MTOC), contributing to the establishment of the cellular polarization and function^9^. The centrosome is geometrically localized adjacent to the nucleus, as its name indicates, and displays a remarkably well-conserved micro-structure among distant organisms^10^. In osteoclasts, electron microscopy has shown the presence of multiple centrosomes gathered in a single structure namely centrosome clustering^11,12^. The organization of a single centrosome clustering in an osteoclast is originated from the centrosomes in its mononucleated precursors^13^. In another words, the centrosomes of precursor cells are inherited into an osteoclast even after they fused each other, and subsequently they are included into a common centrosome clustering. Centrosome clustering is a unique feature of osteoclasts, but not to all multinucleated cells (e.g., granuloma and muscle cells that loses their original centrosomes after their precursors are fused)^12,14^.

The subcellular repositioning of the centrosome forms the MTOC to determine and sustain a polarized cellular structure and function that regulates directed secretion, absorption, and migration, as is well described in differentiated epithelial cells, glandular cells, and immune cells^15,16^. Despite its central role of centrosome in the cellular organization of mononuclear cells, the functional regulation of centrosome clustering in osteoclasts, and its relevance in the pathophysiology of skeletal diseases are not well documented. Contrary to a previous finding which indicated the loss of MTOC clusters upon osteoclastogenesis^17^, we have observed that the establishment and maintenance of centrosome clustering during osteoclast differentiation. This study found that CCR5-mediated signaling is an essential component that coordinates the proper clustering of centrosomes and nuclei, and directional lysosomal trafficking, thus demonstrating that the proper centrosome clustering was tightly associated with the establishment of cellular polarity, as well as the exertion of the bone resorptive function of multinucleated osteoclasts. Furthermore, our gene-disease enrichment analysis and reevaluation of the bone phenotype of *Ccr5*-deficient mice demonstrated that the functional regulation of cellular polarity by CCR5-mediated signaling in osteoclasts is involved in the pathophysiology of bone metabolism.

## Results

### Distinctive morphology of osteoclasts in *Ccr5*-deficient mice

**Figure 1:**
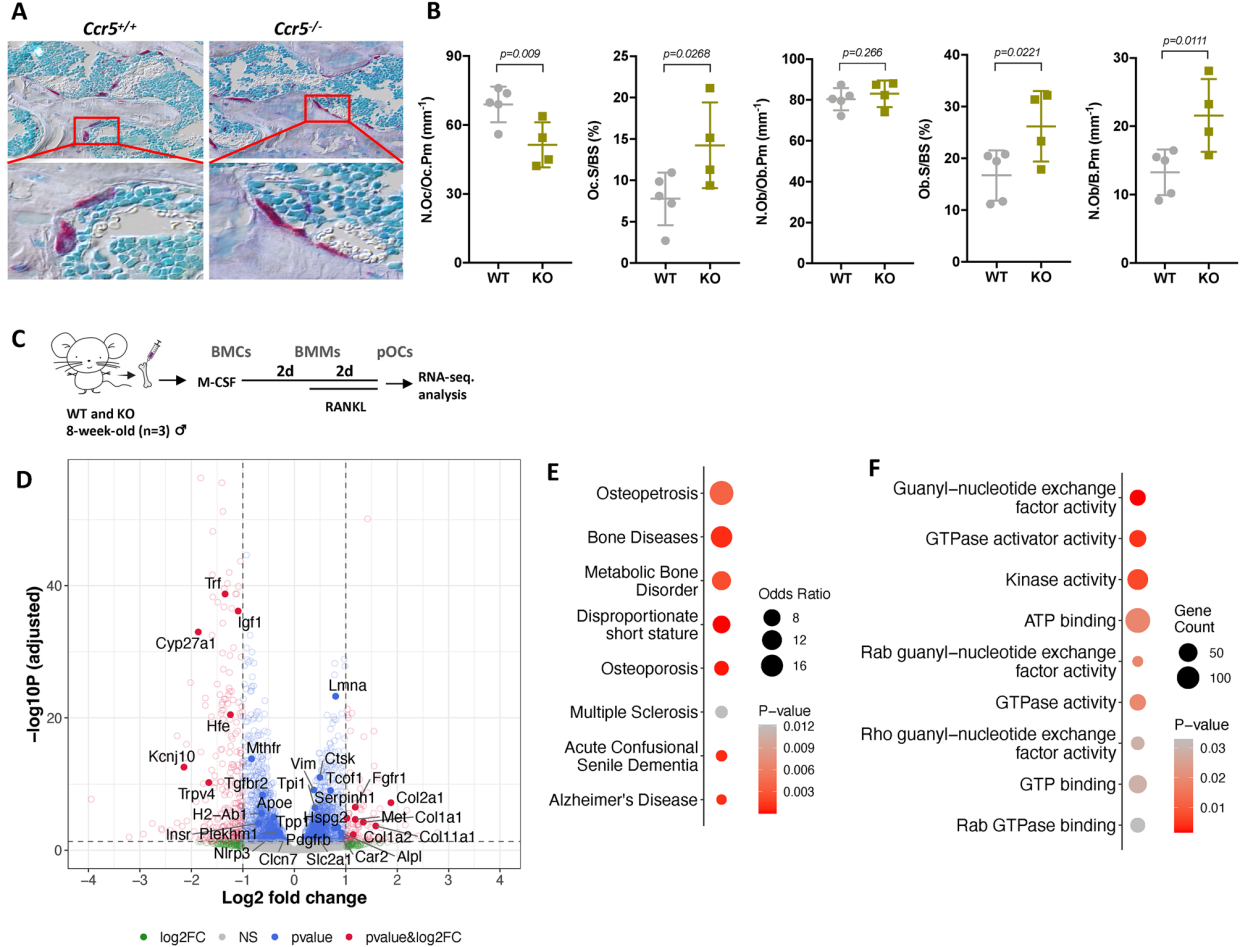
Morphometric phenotype in *Ccr5*-deficient bone and the identification of genes related to human diseases that were differentially expressed in *Ccr5*-deficient osteoclasts. (**A**) Representative histological sections of the distal femurs showing multinucleated TRAP-positive osteoclasts (shown in red). (**B**) A histomorphometric analysis of the distal femur of 10-week-old male mice (WT mice [n = 5] and *Ccr5*-deficienct mice [n = 4]). Data shows the number of osteoclasts per osteoclast perimeter (N.Oc/Oc.Pm), osteoclast surface per bone surface (Oc.S/BS), number of osteoblasts per osteoblast perimeter (N.Ob/Ob.Pm), osteoblast surface per bone surface (Ob.S/BS) and number of osteoblast per bone perimeter (N.Ob/B.Pm). (**C**) The time schedule of osteoclast culture subjected to an RNA-sequencing analysis. (**D**) A volcano plot illustrating the differentially regulated gene expression between WT and *Ccr5*-deficient osteoclasts (adjusted *P*-value < 0.05). The x-axis of the volcano plot shows the log2 fold change of the gene expression levels in WT and *Ccr5*-deficient osteoclasts (positive values indicate genes were upregulated in *Ccr5*-deficient osteoclasts and vice versa). The two dashed vertical lines mark values − 1 (left) and + 1 (right) in log2 fold change. The dashed horizontal line denotes an adjusted *P*-value of 0.05. The genes associated with osteoporosis and related diseases are highlighted with their symbols. (**E**) Significantly enriched disease phenotypes among genes that were differentially expressed between WT and *Ccr5*-deficient osteoclasts. The gene-disease associations as reported in the DisGeNET database were used. The color and size of the node represent the significance (*p*-value) and the number of genes associated with disease phenotypes, respectively. (**F**) Genes with a log2 (fold-change) value of < 0 and an adjusted *P*-value of < 0.05 were tested for enrichment using DAVID/EASE (ver.6.8). Gene ontology (GO) terms were enriched among the downregulated genes encoding the molecular function. The color of the node represents the significance (*P*-value), and the size of the node represents the number of downregulated genes associated with the GO terms.

Previously, we found that *Ccr5*-deficient mice were tolerant to RANKL-induced bone loss with a distinctive morphology of osteoclasts marked by their substantial cell size and flattened appearance in these mice^18^. In the current study, we systematically compared the bone phenotypes of 10-week-old *Ccr5*-deficient mice and their wild type (WT) littermates under non-pathologic condition to elucidate the relevance between morphological changes and cellular function of osteoclast. Histological observation demonstrated irregularly shaped TRAP-positive osteoclasts (stained in red) in *Ccr5*-deficient bone in comparison to WT bone (Fig. 1A). Consistently, histomorphometry analyses showed a reduced number of osteoclast per osteoclast perimeter (N.Oc/Oc. Pm) indicating a single osteoclasts covering larger bone resorptive surface, and an increased osteoclast surface per bone surface (Oc. S/BS) in *Ccr5*-deficient femurs (Fig. 1B). A micro-CT analysis demonstrated that femurs obtained from *Ccr5*-deficient mice exhibited relatively increased bone mass (BV/TV), albeit not significantly, in comparison to femurs from WT mice (Sup. Fig. S1A and B). Supporting this observation, the parameters of connective density (Conn-Dens.), trabecular number (Tb.N) and separation (Tb.Sp) showed significantly higher scores in *Ccr5*-deficient femurs in comparison to WT femurs. The osteoblast surface per BS (Ob.S/BS) and the number of osteoblasts per bone perimeter (N.Ob/B.Pm) were significantly increased in *Ccr5*-deficient femurs, not in the number of osteoblast per osteoblast perimeter (N.Ob/Ob.Pm). However, the mineral apposition rate (MAR), bone formation rate per BS (BFR/BS) and BFR per bone volume (BFR/BV), which represent the volumetric rate of new bone formation, were comparable between WT and *Ccr5*-deficient femurs (Sup. Fig. S1C and D). Similarly, the femurs of 25-week-old *Ccr5*-deficient aged mice showed significantly increased Conn-Dens and Tb.N values, and decreased Tb.Th and Tb.Sp values (Sup. Fig. S1E and F). Next, we performed an in vitro assessment of osteoclastogenesis using osteoclasts isolated from bone marrow macrophages with *Ccr5*-deficient mice. This assessment included evaluating the number of osteoclasts per well, the number of nuclei per cell, and cell size. (Sup. Figure S2A–D). Consistent with histomorphometry analysis, the cell size was notably larger than in *Ccr5*-deficient osteoclasts, matching what was observed in histology, while the number of osteoclasts per well and nuclei per cell were comparable. Additionally, bone resorption capacity showed significant reduction in *Ccr5*-deficient osteoclasts (Sup. Fig. S2E and F). These findings demonstrated that *Ccr5*-deficient mice showed an osteoclast-rich but not bone volume-reduced phenotype, possibly due the impairment of the bone resorptive functions of their osteoclasts, which is associated with their dysregulated cellular structure.

### The transcriptomic signature of *Ccr5*-deficient osteoclasts

We conducted a transcriptome analysis of osteoclast lineage cells from *Ccr5-*deficient mice. Obtained osteoclast precursor cells (pOCs) were subjected to RNA-sequencing (Fig. 1C). A differential expression analysis of RNA-sequencing identified 2,900 genes that showed significantly altered expression levels in *Ccr5-*deficient mice in comparison to WT mice (Fig. 1D). To investigate the association of the gene expression profiles from *Ccr5*-deficient mice with the genetic information of human diseases, we conducted a functional enrichment analysis based on the sets of genes associated with various human disease phenotypes^19^. Among the highly enriched phenotypes were diseases related to osteoclast malfunction, such as osteoporosis and metabolic bone disorders (Fig. 1E). A molecular function analysis from the Gene Ontology (GO) database demonstrated that the GTPase activity, GTP- and ATP binding-related genes were significantly downregulated in *Ccr5*-deficient osteoclasts (Fig. 1F and Sup. Table S1). We previously reported that the blockade of CCR5 was not associated with significant changes in the mineralization or expression levels of *RUNX2, SP7* and *ALP* in osteoblastic differentiation of human mesenchymal stem cells^18^. To find the rationale for the increased Tb.N values in vivo, we investigated the coupling factors from osteoclasts in RNA sequencing data (Sup. Figure S3A). The transcriptional expression of *Tgfb3* and *Efnb1* was significantly increased, while that of other factors (e.g., *Igf1, C3, Nrp1* and *Fas*) was markedly decreased in *Ccr5*-deficient osteoclasts in comparison to WT osteoclasts. Additionally, we have investigated the coupling factors in co-culture system with osteoclasts and osteoblasts (Sup. Figure S3B). The transcriptional expression of *Igf1, C3* and *Nrp1* were significantly increased in *Ccr5*-deficient co-culture system. These differences in gene expression between in vitro and in vivo settings can be attributed to variations in the cellular environment, interactions among cells, immune responses, and temporal/spatial factors.

### Impaired nuclear clustering in *Ccr5*-deficient osteoclasts

**Figure 2:**
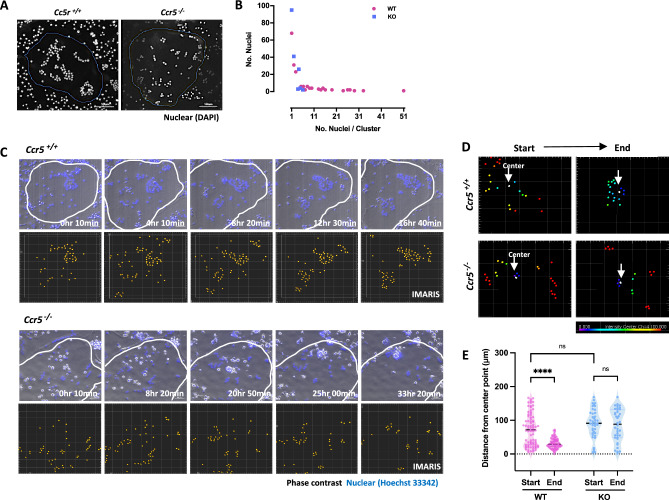
Disintegrated nuclear clustering in *Ccr5*-deficient osteoclasts. (**A**) Nuclear clustering in WT and *Ccr5*-deficient osteoclasts. Representative confocal images of a single mature osteoclast of each genotype are shown. Nuclei were stained with DAPI (shown in white). Scale bar, 100 μm. (**B**) Quantification of the number of nuclei/cluster (n = 6 biological replicates, each from WT and KO). (**C**) Time-lapse live cell imaging of nuclear clustering during maturation of WT and *Ccr5*-deficient osteoclasts. The representative time-course of fluorescence and corresponding phase-contrast images are merged and shown in the upper panels. A tracking analysis of nuclei of the same images is shown. Scale bar, 50 μm. (**D**) The measurement point analysis of nuclei. The distance of each nucleus from the center point (indicated by white arrows) is shown in a graded colors of spectrum. (**E**) Violin plots of the tracked distance of nuclei. The number of analyzed nuclei were as follows: start = 81, end = 6 in WT, start = 42, end = 38 in *Ccr5*-deficient osteoclasts.

We noticed that the subcellular positioning of their nuclei of cultured *Ccr5*-deficient osteoclasts was obviously impaired (Fig. 2). Nuclear plotting analyses demonstrated that nuclear clusters in WT osteoclasts contained 20–30 nuclei per cluster on average, whereas *Ccr5*-deficient osteoclasts mostly formed nuclear clusters containing 5–10 nuclei per cluster (Fig. 2A, B). We next monitored nuclear movements during the differentiation of osteoclasts by time-lapse imaging (Fig. 2C and Sup. Mov. 1). In WT osteoclasts, clustering of nuclei was obviously observed during the course of differentiation. In contrast, multiple scattered nuclei in *Ccr5*-deficient osteoclasts moved to the cellular periphery without clustering. This impaired nuclear clustering in osteoclasts was quantitatively confirmed by a spatial analysis of nuclear movement (Fig. 2D, E).

### Disrupted centrosome clustering and microtubule anchoring in *Ccr5*-deficient osteoclasts

**Figure 3:**
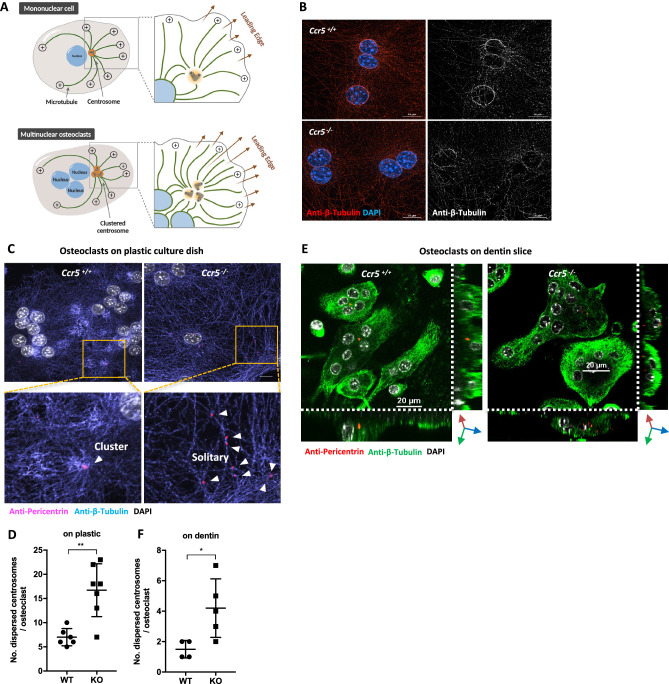
Impaired centrosome clustering in *Ccr5*-deficient osteoclasts. (**A**) Schematic drawings of a centrosome-based MTOC and clustered centrosome-based MTOCs in mono-nuclear cells and multi-nuclear osteoclasts, respectively. (**B**) A super-resolution imaging analysis of microtubules surrounding nuclei in WT and *Ccr5*-deficient mature osteoclasts. Microtubules were visualized by immunofluorescence with anti-β-Tubulin (shown in red, left panels). The maximum intensity projection of reconstructed 3D is shown. Nuclei were stained with DAPI (shown in blue). Gray scale images of β-Tubulin are also shown in right panels. Scale bars; 10 μm. (**C**) An imaging analysis of the centrosome clustering in WT and *Ccr5*-deficient mature osteoclasts cultured on plastic dishes. Representative confocal images of immunofluorescence staining with anti-Pericentrin (shown in magenta) and anti-β-Tubulin (in blue). Nuclei were stained with DAPI (shown in white). Images were visualized with maximum intensity projection after 3D reconstruction. Magnified views of rectangle areas indicated in upper images are shown in bottom panels. Scale bars: 10 μm. (**D**) Quantified data of number of dispersed centrosomes per osteoclasts on plastic. Biological replicates, each from WT (n = 6) and KO (n = 7). (**E**) A 3D imaging analysis of the centrosome clustering in WT and *Ccr5*-deficient mature osteoclasts cultured on dentin slices. Representative 3D reconstructed confocal images of immunofluorescence staining with anti-Pericentrin (shown in red) and anti-β-Tubulin (in green). Nuclei were stained with DAPI (shown in white). The intracellular distribution of centrosomes was demonstrated in a 3D manner along XYZ axes. (**F**) Quantified data of number of dispersed centrosomes per osteoclasts on dentin. Biological replicates, each from WT (n = 4) and KO (n = 5).

In mononuclear cells, the centrosome is located adjacent to nucleus, thus establishing its MTOC that is critical for the cellular polarity. Multinuclear osteoclasts establish their unique MTOCs by the centrosome clustering inherited from their mononuclear precursors during their differentiation (Fig. 3A). We observed and compared the centrosome clustering and its associated microtubule network between WT and *Ccr5*-deficient osteoclasts on plastic culture dishes (Fig. 3B, C). In WT osteoclasts, microtubules (marked by β-Tubulin) encircled cellular nuclei, and tightly anchored to a cluster of centrosomes (marked by Pericentrin) (Fig. 3B, C [arrowheads], respectively). However, in *Ccr5*-deficient osteoclasts, the association of microtubules to cellular nuclei was disrupted, and multiple isolated centrosomes were seen (Fig. 3D). These observations were also confirmed in cells on dentin slices (Fig. 3E, F), and were therefore irrespective of the surface to which they were attached. These findings suggested that the establishment of MTOC, and thus the structural cellular polarity in *Ccr5*-deficient osteoclasts was disrupted.

### Abnormal localization of lysosomes and impaired Cathepsin K secretion but not synthesis in *Ccr5*-deficient osteoclasts

**Figure 4:**
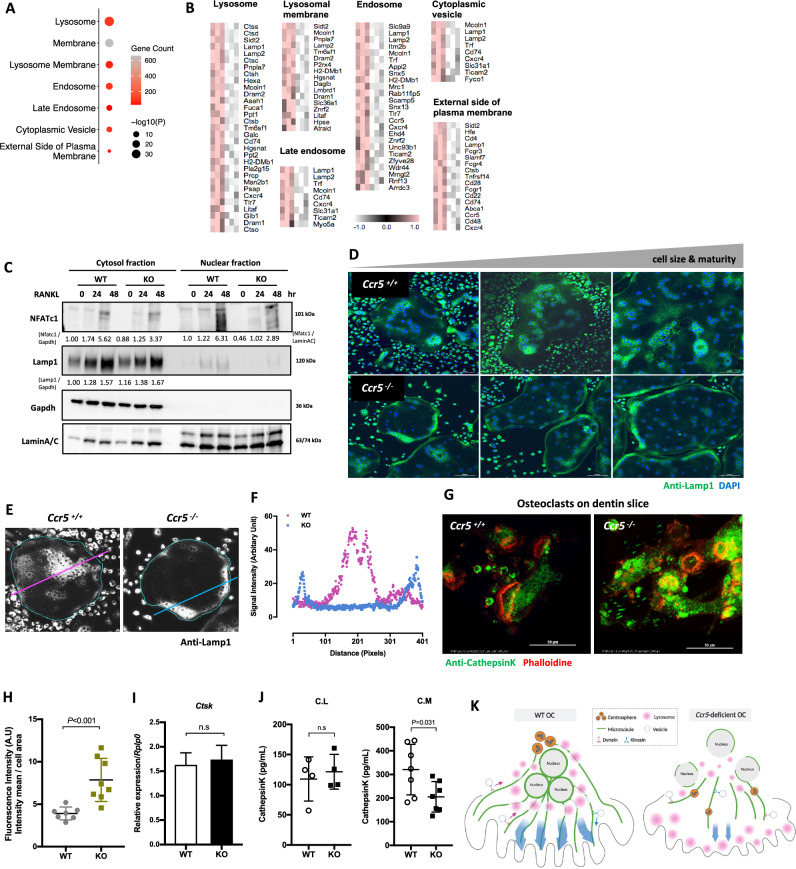
Altered lysosomal localization and impaired Cathepsin K secretion in *Ccr5*-deficient osteoclasts. (**A**) Gene ontology (GO) terms for cellular components that are enriched among the downregulated genes. The color of the node represents the number of genes, and the size of the node represents the significance of enrichment. (**B**) Heatmaps of representative genes associated with cellular membrane, lysosome and vesicle signaling are shown. (**C**) The time-course immunoblotting analysis of Nfatc1 and Lamp1 in differentiating osteoclasts after RANKL stimulation. WT and *Ccr5*-deficient cells were stimulated by RANKL for the indicated times. Cell lysis was divided into cytosol and nuclear fractions, and each fraction was normalized by Gapdh and LaminA/C, respectively. Quantification of immunoblotting data were measured by ImageJ and indicated under the images. All full-length uncropped original western blots are included in a Sup. Fig. S7. (**D**) The cellular distribution of Lamp1 in WT and *Ccr5*-deficient mature osteoclasts. Cells were subjected to immunofluorescence staining with anti-Lamp1 (shown in green) with counterstaining of nuclei with DAPI (shown in blue). Three distinct sizes of osteoclasts are shown for maturity-dependent comparisons. Scale bars: 100 μm. (**E**, **F**) The quantitative analysis of Lamp1 distribution across cells. The fluorescence intensity derived from Lamp1 was plotted along lines shown in (**F**). (**G**) Cellular localizations of Cathepsin K and actin rings (shown in green and red, respectively) in WT and *Ccr5*-deficient osteoclasts cultured on dentin slices. Scale bars: 50 μm. (**H**) The fluorescence intensity analysis of the Cathepsin K expression in WT and *Ccr5*-deficient osteoclasts, conducted after obtaining 3D confocal images. (**I**) The transcriptional expression of *CtsK* in WT and *Ccr5*-deficient osteoclasts. (**J**) The concentration of Cathepsin K in cell lysates (C.L) and conditioned medium (C.M) in WT and *Ccr5*-deficient osteoclasts. (**K**) Schematic drawings of the cellular polarity and vesicle trafficking in WT and *Ccr5*-deficient osteoclasts. In WT osteoclasts, centrosomes were well gathered to form a clustered centrosome adjacent to a nuclear cluster, thus establishing clustered MTOCs. Concomitantly, lysosomal vesicles were predominantly located surrounding the nuclear cluster. Therefore, the microstructural and functional cellular polarity was established, through which the regulation of inward and outward vesicle trafficking can be achieved. In *Ccr5*-deficient osteoclasts, the centrosome clustering and the MTOC formations were impaired, while lysosomal vesicles were predominantly located at the cellular periphery, indicating abrogated vesicle trafficking.

These findings at subcellular structure prompted us to compile a comprehensive catalog of functional cellular components from the GO databases (Fig. 4A, B). This analysis demonstrated that the lysosome-, endosome- and membrane-related genes were significantly downregulated in *Ccr5*-deficient osteoclasts. To validate the lysosome-related defects predicted by the transcriptomic signature of *Ccr5*-deficient osteoclast, we examined the expression of osteoclast-related proteins by immunoblotting (Fig. 4C). The protein level of NFATc1, a master transcription regulator of osteoclast differentiation, was markedly increased in cytosol upon RANKL stimulation and subsequently translocated to the nuclei in WT cells, but was markedly decreased in *Ccr5*-deficient cells. The lysosomal-associated membrane protein 1 (Lamp1) level was also simultaneously increased with RANKL treatment, whereas no obvious changes in Lamp1 levels were observed between WT and *Ccr5*-deficient osteoclasts. We then investigated the cellular distribution of Lamp1 during osteoclast maturation, marked by its cellular size, by immunofluorescence (Fig. 4D). Lamp1-derived fluorescence signals were accumulated into several clusters apart from the cellular margins in maturating WT osteoclasts, whereas these signals in *Ccr5*-deficient osteoclasts were mostly observed along the inside of the cellular rim (Fig. 4D). A fluorescence intensity plotting analysis against Lamp1 confirmed this observation (Fig. 4E, F). Notably, the enriched cellular expression of Lamp1 was detected in juxtanuclear position in both WT and *Ccr5*-deficient osteoclasts, suggesting that cellular positionings of lysosomes as well as nuclei were disrupted by *Ccr5-*deficiency during osteoclastogenesis. To see whether or not dysregulated lysosome trafficking and impaired cellular polarity in *Ccr5*-deficient osteoclasts affected the secretion process of bone-degrading enzyme, we observed and compared the cellular colocalization of Cathepsin K (Ctsk), a representative enzyme for bone resorption, and Lamp1 in WT and *Ccr5*-deficient osteoclasts cultured on plastic culture dishes and dentin slices (Fig. 4G and Sup. Fig. S5A, respectively). Ctsk and Lamp1 were well colocalized in both genotyped cells (Sup. Fig. S4A). Intense Ctsk- and Lamp1-derived fluorescence signals were observed around nuclear clusters in WT cells, whereas they were observed in peripheral sites in *Ccr5*-deficient cells (Sup. Fig. S5A). On dentin slices, WT cells showed well encircled actin rings (in red) with faint fluorescence signals from Ctsk (in green), whereas *Ccr5*-deficient cells showed actin ring destruction with relatively intense signals from Ctsk (Fig. 4G). The quantification of fluorescence signals / cell area indicated the significantly higher expression of Ctsk in *Ccr5*-deficient osteoclasts in comparison to WT osteoclasts (Fig. 4H). Although the transcriptional levels of *Ctsk* and the cellular concentration of Ctsk were indistinguishable between WT and *Ccr5*-deficient osteoclasts, the concentration of Ctsk in conditioned medium (CM) was significantly decreased in *Ccr5*-deficient osteoclasts (Fig. 4I, J). These results suggested that the lysosomal secretion of Ctsk but not its synthesis was impaired in *Ccr5*-deficient osteoclasts (Fig. 4K).

### Rescue of cellular polarity and Ctsk secretion in *Ccr5*-deficient osteoclasts by constitutive activation of the signaling of Rho or Rac GTPases

**Figure 5:**
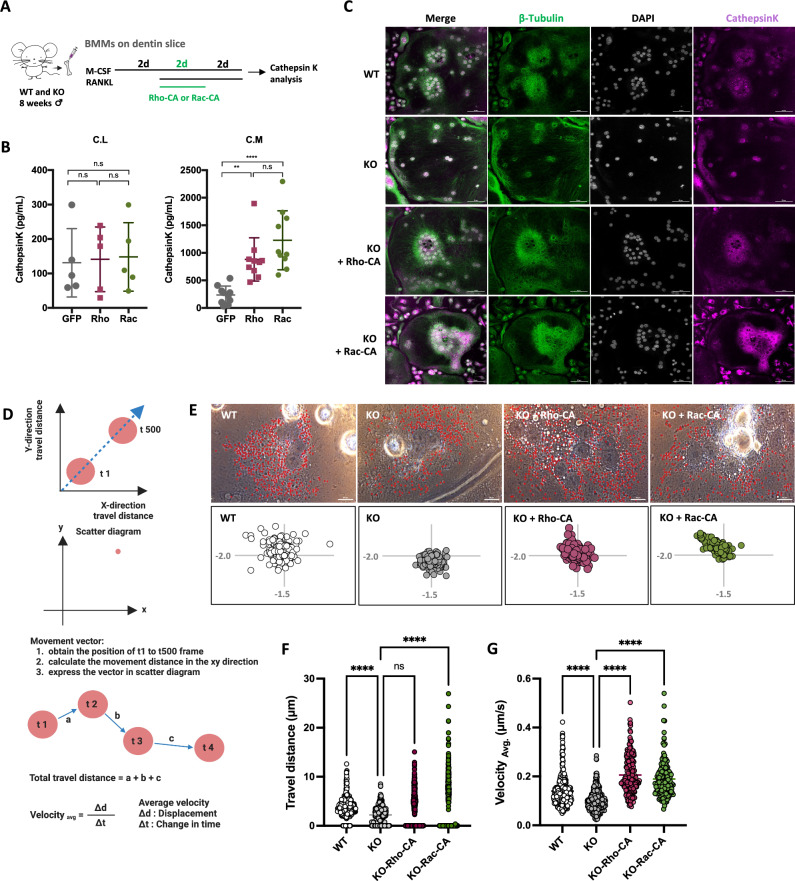
Rescue of impaired MTOC and lysosomal vesicle trafficking in *Ccr5*-deficient osteoclasts by the expression of constitutively active forms of Rho or Rac. (**A**) The experimental schedule of adenovirus-mediated gene transfer for forced expression of constitutively active forms of Rho or Rac (Rho-CA or Rac-CA) during the differentiation of osteoclast cultured on dentin slices. (**B**) Cathepsin K concentrations in cell lysates (C.L) and conditioned medium (C.M) measured by an ELISA assay. *P < 0.05 by Student’s *t*-test. The data are shown as the mean ± SD. (**C**) The recovery of nuclei clustering and the subcellular localization of β-tubulin and Cathepsin K by the overexpression of Rho-CA or Rac-CA in *Ccr5*-deficient osteoclasts. Representative images of immunofluorescence staining with anti-β-Tubulin (shown in green) and anti-Cathepsin K (shown in magenta) in mature osteoclasts derived from WT and *Ccr5*-deficient bone. (**D**) The workflow of quantification for intracellular vesicle trafficking. (**E**) Representative images of lysosomal vesicle trafficking obtained using an external phase contrast microscope (upper panels). Tracked vesicles are marked in red. Vectors in scatter diagrams showing vesicle movement and directionality are shown in the lower panels. (**F**, **G**) The quantitative analysis of total travel distance (μm) and average of velocity (μm/s) variation, as determined by vesicle tracking. Values indicate the mean ± SD.

In our previous study, impaired actin ring formation of *Ccr5*-deficient osteoclasts was rescued by the forced expression of constitutively active (CA) forms of Rho or Rac (Rho-CA, or Rac-CA)^18^. Our GO enrichment analysis in this study showed that various signals related to small GTPases such as Rho and Rac were significantly downregulated in *Ccr5*-deficient osteoclasts (Sup. Table S1). This study examined whether or not the expression of Rho-CA, or Rac-CA also rescued the impaired cellular polarity and Ctsk secretion in *Ccr5*-deficient osteoclasts. To measure the secretion of Ctsk, we seeded the bone marrow cells onto dentin slices and further differentiated into mature osteoclast (Fig. 5A). A quantization of Ctsk release of mature osteoclasts showed that the overexpression of either Rho-CA or Rac-CA in *Ccr5*-deficient osteoclasts led to the significant recovery of impaired Ctsk secretion in the CM (Fig. 5B). However, the concentrations of intracellular Ctsk (cell lysates, CL) among the groups were comparable to those in control (GFP-vehicle). Consistently, immunofluorescence studies demonstrated that the overexpression of either Rho-CA or Rac-CA in *Ccr5*-deficient osteoclasts also rescued nuclear clustering, microtubule anchoring, and cellular localization of Ctsk (Fig. 5C). We further investigated lysosomal vesicle trafficking in osteoclasts using an external phase contrast microscope (Fig. 5D–G). The directional movement of lysosomal vesicle located adjacent to nuclei was monitored in a time-lapse manner (Sup. Movie 2). Directional movement of the vesicles, predominantly toward the nucleus, was observed in WT osteoclasts, while most of the vesicles in *Ccr5*-deficient osteoclasts appeared to stay around their initial positions (Fig. 5E). Consistently, total travel distance and average velocity of the vesicles in KO cells were significantly reduced in comparison to WT osteoclasts (Fig. 5F, G). Notably, the overexpression of either Rho-CA or Rac-CA in KO rescued the directional movement of the vesicles toward nuclei (Fig. 5E). The overexpression of Rho-CA rescued the velocity but not the distance, while the overexpression of Rac-CA rescued both, suggesting a functional distinction between Rho and Rac in vesicle trafficking (Fig. 5F, G). We also performed a comparative analysis to determine whether restoring Ccr5 expression in *Ccr5*-deficient osteoclasts could rescues the impaired centrosome clustering and lysosome localization (Sup. Fig. S5A–E). First, we assessed the *Ccr5* gene expression after overexpressing Adv-Ccr5 by real-time qPCR (Sup. Fig. S5A). Adv-Ccr5 overexpression was effectively and significantly increased *Ccr5* gene expression levels compared to *Ccr5*-deficieny. Consistent with time schedule for Rho-CA and Rac-CA treatment (Fig. 5A), Adv-Ccr5 was treated into osteoclast precursors 2 days after RANKL stimulation. The increased dispersed centrosome per osteoclast was significantly reduced with Ccr5 overexpression (Sup. Fig. S5B and C), and lysosome localization was observed to relocated from the periphery to an intracellular region closer to near nuclei clustering (Sup. Fig. S5D, E). Collectively, these results suggested that the cellular polarity and lysosomal trafficking in osteoclasts were regulated by Ccr5-mediated signaling through small GTPases such as Rho and Rac.

### Blockade of CCR5 downregulates the *PLEKHM1* expression in human osteoclasts

**Figure 6:**
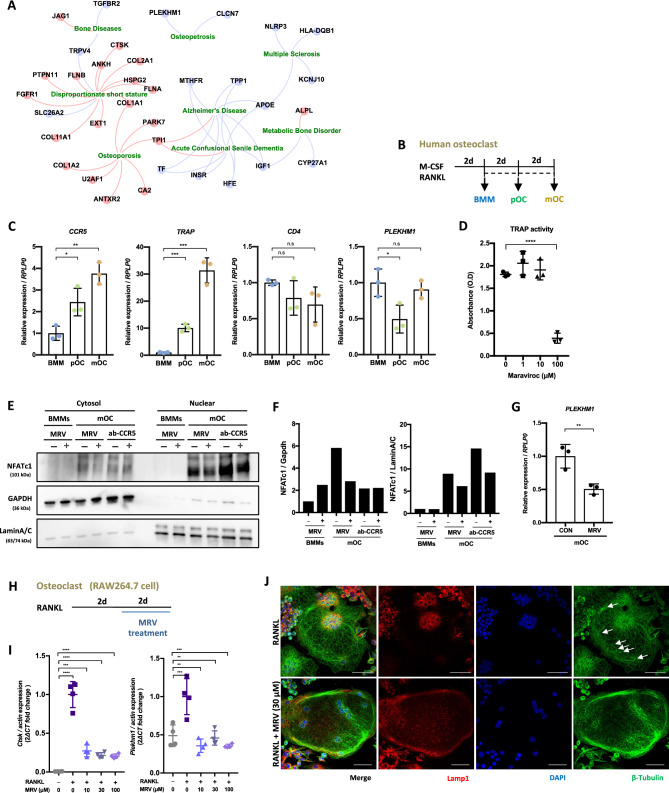
Phenocopy of mouse *Ccr5*-deficient osteoclasts by blockade of CCR5 in human osteoclasts. (**A**) Disease phenotypes significantly enriched among genes that were differentially expressed between WT and *Ccr5*-deficient osteoclasts. The gene-disease associations reported in the DisGeNET database were used. Diseases and genes are respectively shown as rectangles and circles. Upregulated and downregulated genes in *Ccr5*-deficient osteoclasts in comparison to WT osteoclasts were marked in red and blue, respectively. (**B**) Time schedule of cultured human osteoclast differentiation. Human osteoclasts were cultured with M-CSF and RANKL for 6 days. Bone marrow macrophage (BMM), osteoclast precursor (pOC) and mature osteoclast (mOC) were defined as shown. (**C**) The transcriptional expressions of *TRAP, CCR5, CD4* and *PLEKHM1* measured by qPCR. (**D**) The effect of Maraviroc (MRV) treatment on TRAP activity. (**E**) The effects of MRV (100 μM) and neutralizing anti-CCR5 antibodies (10 μg/mL) on the expression and nuclear localization of NFATc1, as determined by an immunoblotting analysis. (**F**) The quantification of immunoblotting data measured by ImageJ. All full-length uncropped original western blots are included in a Sup. Fig. S8. (**G**) The transcriptional expression of *PLEKHM1* in mature human osteoclasts measured by qPCR. (**H**) The cell culture and MRV treatment schedule of RAW264.7 cells. RAW264.7 cells were cultured with RANKL (50 ng/mL) for 48 h. Then, MRV was added and cells were cultured for another 2 days at the indicated concentration. (**I**) The transcriptional expressions of *Ctsk* and *Plekhm1* in RAW264.7 cells analyzed by qPCR. (**J**) Effect of MRV on the subcellular localizations of Lamp1 and β-Tubulin by immuno-staining with anti-Lamp1 (shown in red) and anti-β-Tubulin (in green). All data are shown as the mean ± SD. *P* values were determined by an unpaired one-way ANOVA.

Our human disease and gene network analysis suggested a link between *CCR5* and *PLEKHM1* genes in the context of osteopetrosis (Fig. 6A). A missense mutation in *PLEKHM1* has been reported to cause osteopetrosis^22^ due to impaired vesicle trafficking of osteoclasts^23^. The *PLEKHM1* gene has been reported to be functional in vesicle trafficking of osteoclasts^23^. To determine whether *PLEKHM1* is regulated by CCR5 in human osteoclast differentiation, we cultured and differentiated human osteoclasts with or without Maraviroc (MRV), an inhibitor of CCR5. Human BMMs were stimulated with M-CSF for 2 days and differentiated into pOC and mOC with RANKL for further 2 and 4 days, respectively (Fig. 6B). In this culture without MRV, the transcriptional expression levels of *CCR5* and *TRAP* were gradually upregulated as cells differentiated, while the level of *CD4*, a marker of immunoglobulin to monitor changes in response to osteoclastogenesis, was comparable throughout the culture periods (Fig. 6C). The *PLEKHM1* expression level was significantly downregulated in pOC, but was then upregulated to the initial expression level in mOC. Treatment with either MRV or CCR5-neutralization antibodies (ab-CCR5) decreased the nuclear localization of NFATc1 in mOC (Fig. 6E, F). MRV treatment also significantly reduced the TRAP activity and transcriptional level of *PLEKHM1* (Fig. 6D, G)*.* These findings suggested that CCR5-mediated signaling was required for human osteoclast differentiation, including the regulatory expression of *PLEKHM1.* To confirm whether or not MRV treatment phenocopied *Ccr5* deficiency in mouse osteoclasts, we treated differentiating mouse RAW 264.7 cells with MRV (Fig. 6H). Consistently, the *Ctsk* and *Plekhm1* expression levels were significantly downregulated by MRV treatment (Fig. 6I). MRV treatment in mouse cultured osteoclasts induced spatially impaired nuclear clustering associated with irregular tubulin anchoring and Lamp1 distribution, as was observed in *Ccr5*-deficient osteoclasts (Fig. 6J). Taken together, these results suggested that CCR5-mediated signaling was essential for the differentiation and function of osteoclasts through the regulation of their cellular polarity in both human and mouse cells, which included the regulatory expression of the *Plekhm1* gene, which is essential for vesicle trafficking of osteoclasts, and bone metabolism.

## Discussion

The current study discovered aberrant positioning of nuclei and lysosomes in *Ccr5*-deficient mature osteoclasts, which was associated with defects in the centrosome clustering and microtubule network. Microtubule organization supports critical cellular functions, including assembly of the mitotic spindle apparatus during cell division, establishment of cell polarity, and by serving as a conduit for intracellular trafficking^24^. The MTOC in animal cells is the centrosome, which organizes microtubule arrays from a supramolecular matrix called the pericentriolar material (PCM). Unlike myoblast-derived multinucleated myotubes in which centrosomes are eliminated after exit from the cell cycle and upon differentiated, monocyte-derived multinucleated osteoclasts keep all centrosomes associated with their respective nuclei^25^. In post-cell fusion of osteoclasts, centrosomes were observed to aggregate and form their clusters that were consistently associated with large radial microtubule arrays. This clustering of centrosome is a unique phenotype not observed in other multinuclear cells; thus, the centrosome clustering is a specific subcellular structure of osteoclasts. In our observation, the centrosome clustering in WT osteoclast linked with dense microtubule network were predominantly localized vicinity to the clustered nuclei of mature osteoclasts, and lysosomes were concentrated around them. On the contrary, numbers of nuclei were scattered in the cellular periphery, and a lot of solitary centrosomes with thin microtubules were observed in *Ccr5*-deficient osteoclasts. Accordingly, a dispersed pattern of lysosomes in the cellular periphery was observed in mature *Ccr5*-deficient osteoclasts, while fully differentiated WT osteoclasts showed lysosomal accumulation around the clustered nucleus. Therefore, MTOC was destructed in *Ccr5*-deficient osteoclast.

The inhibition of the centrosome clustering reduces the actin ring size and decreases the bone resorptive function in osteoclasts^13^. Since large radial microtubule arrays have been previously observed within the sealing zone, a critical cellular microstructure for bone resorption^6^, it is possible that centrosome clustering can constitute large radial microtubule networks that mediate bone resorption. Cathepsin K, which is the principal lysosomal acidic hydrolase degrading the organic matrices of bone, is secreted into the resorption lacunae in resorbing osteoclasts. We observed that the extracellular secretion of Ctsk was significantly decreased in *Ccr5-*deficient osteoclasts in comparison to WT cells, which was likely to be associated with the distinct distribution of Ctsk-containing lysosomes between *Ccr5-*deficient and WT osteoclasts. It is possible that proper centrosome clustering and the establishment of MTOCs in osteoclasts enables efficient lysosome trafficking across the cell, thus establishing functional cell polarity as a single centrosome does in mononuclear cells. Impaired microtubule nucleation, and dispositioning of nuclei in *Ccr5*-deficient osteoclasts made us imagine that multiple MTOCs provides structural basis for mechanical forces that efficiently push and pull nuclei to support their proper location, and vice versa. Large osteoclasts possibly need their multiple MTOC to maintain their huge cell body, motility, and bone-resorbing function. Therefore, our observation suggested that CCR5 is essential for establishing functional cell polarity in osteoclasts.

We previously reported that *Rac1* and *RhoA* were downregulated in Ccr5-deficient osteoclasts, and the activation either of Rho or Rac in *Ccr5*-deficient osteoclasts recovered disrupted actin ring formation, a landmark structured for ruffled border formation. In this study, we further observed that the same experiments also recovered positioning of nuclei and secretion of Ctsk as well as lysosomal directionality. Therefore, Ccr5-mediated activation of these small GTPase regulates multiple MTOC formation and functional cell polarity in osteoclasts. Thus, elucidation of cytoskeletal abnormality and osteolytic dysfunction due to *Ccr5*-deficiency in this study likely provides insights not only into osteoclast biology, but in lysosome biology as well, especially in related functions in centrosome and lysosome dynamics.

The comparison between the differentially expressed genes in *Ccr5*-deficient osteoclasts and the list of human disease-associated genes indicated that genes regulated under *Ccr5* were highly associated with metabolic bone disorders, including osteopetrosis. The bone phenotype in *Ccr5*-deficient mice was osteoclast-rich with a slightly higher trabecular bone mass in comparison to their WT littermates. We previously reported that *Ccr5*-deficient mice were less susceptible to RANKL-induced osteoporosis due to the functional loss of bone resorption in osteoclasts^18^. The relevance of these mouse studies is supported by a clinical study that compared the bone mineral density of HIV-1 infected patients treated with tenofovir disoproxil fumarate (TDF, a reverse-transcriptase inhibitor) and MRV as initial anti-retroviral treatment for 48 weeks^27^. The MRV-treated group showed significantly less bone loss in the lumbar spine and hip in comparison to the TDF-treated group^28,29^, although TDF has been reportedly associated with decrease in BMD through renal proximal tubule toxicity, resulting in phosphate wasting and increased bone turnover^28,29^. HIV-1 infection in human osteoclasts markedly enhanced their bone resorptive function through the activation of Src signaling, regulating the structure and function of the sealing zone^30^. These findings suggested that the functional modulation of CCR5 in osteoclasts is involved in the pathophysiology of bone metabolism in both mice and humans.

Our gene-disease enrichment analysis comparing *Ccr5*-deficient osteoclasts and human diseases also highlighted two genes: *CLCN7* and *PLEKHM1.* The *CLCN7* gene plays an essential role in the acidification of the extracellular resorption lacunae^31^. Although the differential inhibition of CLCN7 showed that this molecule was also a good candidate mediator of the bone-resorptive function, the results from RNA-seq data revealed no significant differences between WT and *Ccr5*-deficeient mice. Mutation of the *PLEKHM1* gene has been identified as a cause of osteopetrosis in *ia/ia* (incisors absent) rats, as well as a subset of patients with intermediate osteopetrosis, due to diminished bone resorption and an intrinsic defect in ruffled border formation associated with the accumulation of intracellular lysosomes in osteoclasts^23,32^. In vitro mechanistic studies showed that PLEKHM1 regulated the peripheral distribution and secretion of lysosome through a protein complex consisting of PLEKHM1, DEF8, RAB7, FMA98A and NDEL1 (at a minimum)^33^. Plekhm1 binds to Rab7-Arl8b to mediate lysosome fusion^34–36^, and is reported as a dual effector of Rab7 and Arl8b that simultaneously binds these GTPases, bringing about the clustering and fusion of late endosomes and lysosomes from cytosol to the perinuclear region. The deletion of *Plekhm1* led to the accumulation of Lamp1-positive lysosomes at the cell periphery^35^, as was observed in *Ccr5*-deficent osteoclasts. Taken together, these previous observations and the finding of our present study suggest that *Ccr5*-deficiency largely phenocopies *Plekhm1* deficiency, especially in the osteoclast function.

In conclusion, our study has revealed that *Ccr5* plays a crucial role in the centrosome clustering and cellular polarity of osteoclasts, which are necessary for efficient bone resorption in mice. Moreover, our gene-disease enrichment analysis highlighted the association between *Ccr5*-deficient osteoclasts and human diseases indicated that genes under the regulation of Ccr5 are associated with metabolic bone disorders. These findings suggest that CCR5-mediated signaling and its gene regulatory network play a significant role in regulating cellular polarity in osteoclasts (Sup. Fig. S6) and modulating the rate of bone turnover, which provides potential targets for novel druggable approaches to treat skeletal disorders.

## Materials and methods

### Ethical guidelines for animal studies

All the animal experiments conformed to the Institutional Guidelines for the Care and Use of Laboratory Animals in Research and with the approval of the local ethics committees of Ehime University and Hokkaido University (2020-043, 2021-0018). This study is reported accordance with ARRIVE guidelines.

### Mice

*Ccr5*-deficient mice (*Ccr5*^*–/–*^) were generated as previously described^37^. All mice were backcrossed for 8 to 10 generations on the C57BL/6J mouse background. The mice were housed in a specific pathogen-free environment in the animal facilities of Hokkaido University.

### Reagents

Recombinant mouse and human M-CSF and RANKL for osteoclast differentiation were purchased from Wako Pure Chemical Industries, Ltd. (Osaka, Japan). Maraviroc (S2003) was purchased from Selleck Chemicals (Houston, TX). Human neutralizing CCR5 antibody (MAB182) was purchased from R&D Systems. The ELISA kit for Cathepsin K was provided by Cloud-Clone Corporation (Houston, TX). AlexaFluor 488 (A12379) and 568 (A12380) phalloidin, which were used for actin structure staining were purchased from Invitrogen Thermo Fisher Scientific (Waltham, MA). Nuclei were stained using 4’,6-diamidino-2-phenylindole (DAPI) (Dojindo, Kumamoto, Japan). The antibodies used for immunoblotting and immunohistochemistry were as follows: β-actin (A5316) from Sigma-Aldrich (St. Louis, MO); GAPDH (#2118), LaminA/C (#4777) from Cell Signaling Technology (Danvers, MA); NFATc1, Lamp1 (ab24170), Cathepsin K (ab19027), Pericentrin (ab4448) and Tubulin (ab6160) from Abcam (Cambridge, MA). The secondary antibodies Alexa488 (rabbit A11008, mouse A11029), 568 (rabbit A11011, mouse A11004) and 47 (rabbit A21245, mouse A21236, rat A21247)-labeled IgG (all dilution 1:1000) were purchased from Molecular Probes / Invitrogen Thermo Fisher Scientific. Calcein was purchased from Sigma-Aldrich. Mouse Ccr5 (Adenoviral) vector was generated from VectorBuilder (Chicago, IL).

### Cultures of mouse and human osteoclasts

Freshly harvested mouse bone marrow cells (BMCs) isolated from 8 to 10-week-old mice cultured in α-MEM (Gibco BRL, Gaithersburg, MD) were used as the source of osteoclasts. Bone marrow-derived macrophages (BMMs) were induced with M-CSF (50 ng/mL) in a 6 cm dish with 15–18 h of incubation and differentiated into osteoclast with RANKL (50 ng/mL) for an additional 5–6 days. The culture media was replaced every 3 days. Normal human osteoclast precursor cells (Poietics™ Osteoclast Precursor Cells System, Cat. No. 2T-110) were purchased from Lonza Walkersville, Inc. (Walkersville, MD), and were cultured with Osteoclast Precursor Cell Basal Medium (PT-8201, Lonza). For differentiation into mature osteoclasts, cells were cultured with Osteoclast Precursor Cell Basal Medium including both M-CSF and RANKL according to the manufacturer’s instructions.

### Osteoclast culture on dentin slice

Bone marrow cells were flushed from the femurs and loaded on dentin slices in medium containing M-CSF and RANKL. After 5 days, the conditioned medium was collected for a Cathepsin K ELISA assay. Mature osteoclasts on dentin slice were lysed with RIPA buffer. The extracted proteins were quantified and used for the Cathepsin K ELISA assay.

### Immunocytochemical staining and imaging by microscopy

For immunostaining, cells were fixed with 4% paraformaldehyde in PBS for 10 min, permeabilized with 0.1% Triton X-100/PBS for 30 min, followed by blocking with 0.1% BSA for 30 min. Cells were stained with the indicated specific Abs. For imaging, a μ-slide chamber dish (Ibidi GmbH, Germany) was used to obtain high resolution. Immunofluorescence images were captured using a laser confocal scanning microscope (A1-ECLIPSE Ti2-E, Nikon, Tokyo) equipped with a X60/1.42 oil immersion objective lens. A wide-field fluorescence microscope was also used to capture images of histologically stained sections (ECLIPSE Ni-E, Nikon, Tokyo) and cell culture plates (BZ-800, Keyence, Osaka). Prior to performing live-cell imaging, cells cultured in the chamber dishes were incubated with Hoechst 33342 (ThermoFisher Scientific) solution for 10 min. Then, the chamber dishes were placed on an onstage incubator equipped with A1-ECLIPSE Ti2-E. Time-lapse sequence images of mature osteoclasts were acquired every 10 min until their disappearance by apoptosis. Super-resolution imaging of fixed samples was performed using an N-SIM (Nikon) microscope equipped with a × 100/1.49 oil immersion objective lens (Nikon) and an Andor iXon + electron multiplying charged-coupled device camera (Andor Belfast, UK). All images were processed for quantification using the NIS-Elements (Nikon), IMARIS (Bitplane, Zurich, Switzerland) and ImageJ software programs.

### Non-staining of lysosome trafficking with an external phase contrast microscope

Lysosome trafficking in living osteoclasts was monitored by a non-staining method using an external phase contrast system (Nikon). An inverted microscope with an external phase contrast system was equipped with an oil immersion objective (Nikon, HP Apo TIRF × 100 oil, NA = 1.49). Live images of mature osteoclasts were acquired at 10 loops per second for 50 s. NIS-Elements General Analysis 3 (Nikon) was used to statistically analyze the tracking parameters of lysosomes, including movement, directional movement, speed and travel distance. In the analysis, the dark and white structure spots were distinguished based on different refractive indices, and typical diameter, contrast and intensity parameters were applied to the dark spots. Then, the dark spots were defined as lysosome-like structures.

### The real-time Q-PCR

Total RNA was obtained with a RNeasy kit (Qiagen, Hilden, Germany) in accordance with the manufacturer’s protocols. Total RNA was reverse-transcribed into cDNA using a QuantiTech Reverser Transcription kit (Qiagen, Hilden, Germany). Real-time quantitative PCR was performed with SYBR Green Master Mix and the mRNA expression was detected in triplicate using an Eco™ Real-Time PCR system (Illumina, SD). The sequences were amplified for 40 cycles under two-step conditions: denaturation at 95 °C for 15 s, and annealing and extension at 60 °C for 60 s. The gene expression levels were compared to the *Rplp0* gene expression levels by the 2^−(Ct)^ method. The qPCR primers sequences for human and mouse used in the study are provided in Sup. Table S2.

### Immunoblotting

For immunoblotting, cytosol and nuclear fractions from osteoclasts were collected using Proteo JET Cytoplasmic & Nuclear Protein Extraction kit (Fermentas Life Sciences, Ontario, Canada) with a phosphatase inhibitor cocktail (Sigma-Aldrich) and a protease inhibitor cocktail (Sigma-Aldrich). Cell fractions were subjected to SDS/PAGE under reducing conditions and blotted onto PVDF membranes (Bio-Rad, Hercules, CA) in semi-dry transfer conditions (Bio-Rad blotting apparatus). After blocking with 5% of bovine serum albumin in TBS-T buffer, the membrane was incubated overnight at 4 °C with a primary antibody, and the detection of specific protein was carried out using enhanced chemiluminescence (Bio-Rad).

### Calcein labeling, micro-CT and histomorphometry analysis

Mice were labeled with calcein (20 mg/kg, intraperitoneal injection) in PBS 2 times for every 2 days prior to sacrifice. A micro-CT (μCT) analysis was performed as previously described^18^, and all samples were analyzed performed in a blinded manner. μCT scanning of the proximal femur was performed using a μCT-35 (SCANCO Medical AG, Bruttisellen, Switzerland) with a resolution of 6 μm, and the parameters were three-dimensionally calculated as previously described^38^. The bone histomorphology of the femur was examined using OsteoMeasure (OsteoMetrics, Decatur, GA) with a light microscope. Femurs were fixed in 4% paraformaldehyde for 2 days, were embedded in glycol methacrylate. Sections (thickness: 5-μm) were stained with Villa-nueva, TRAP and toluidine blue. The measurement terminology and units were as defined by the Nomenclature Committee of the American Society for Bone and Mineral Research^39,40^.

### The RNA sequencing analysis

BMCs were harvested from the tibia of 8 weeks-old male *Ccr5-*deficient mice and WT mice. Cells were induced into BMMs with M-CSF for 2 days, and further differentiated into pOCs with RANKL for 2 days. pOCs were then subjected to RNA-sequencing. The analysis was performed as previously described^18^. Briefly, quality of the extracted RNA was assessed using an Illumina kit (Illumina) according to the manufacturer’s instructions. Illumina TruSeq Standard mRNA Sample Prep Kit set A was used for the library preparation. mRNA sequencing was performed using an Illumina MiSeq Reagent kit V3 150 cycle kit with 75 bp paired-end sequencing with a fragment size of ~ 260 bp, trimmed to 75 bp. The sequencing reads were mapped to mouse reference genome (GRCm39) using Spliced Transcripts Alignment to a Reference (STAR, version 2.7.8a)^41^. The aligned reads were quantified into read count data using HTseq-count^42^. The R package *DESeq2*^43^ was used to perform a differential expression analysis, including normalization of read counts and statistical tests. The gene annotation file from the GENCODE project (v27)^44^ was used throughout the analysis. A heatmap was obtained using MeV and KEGG pathway analyses, which were performed using DAVID Bioinformatics Resources version 6.8. Gene-disease associations reported in DisGeNET (v7.0)^45^ were used for a disease enrichment analysis of differentially expressed genes, which was performed with the R package *disgenet2r*^19^.

### Statistical analyses

Data were presented as the mean ± standard deviation (SD) of the indicated number of times an examination was repeated. Statistical significance was determined using the GraphPad Prism 7 (GraphPad Software Inc. La Jolla, CA) software program. The data were analyzed using an unpaired *t-* test (two conditions) and a one-way or two-way ANOVA of variance for multiple comparisons (more than two conditions). Asterisks indicate significant upregulation or downregulation (*P < 0.05, **P < 0.01, ***P < 0.001, ****P < 0.0001). NS indicates that a result was not significant.

### Supplementary Information

Supplementary Information 1.

Supplementary Information 2.

Supplementary Video 1.

Supplementary Video 2.

Supplementary Video 3.

Supplementary Video 4.

Supplementary Video 5.

Supplementary Video 6.

## Data Availability

The RNA-sequencing data of mouse wild-type and knock-out mice are deposited at Gene Expression Omnibus (GEO, Accession Number: GSE226793) and accessible online. Raw data that support the findings of this study are available from the corresponding author, upon reasonable request.

## References

[1] Suda T, Nakamura I, Jimi E, Takahashi N (1997). Regulation of osteoclast function. J Bone Miner Res.

[2] Boyle WJ, Simonet WS, Lacey DL (2003). Osteoclast differentiation and activation. Nature.

[3] Dogterom M, Koenderink GH (2019). Actin-microtubule crosstalk in cell biology. Nat Rev Mol Cell Biol.

[4] Goldmann WH (2018). Intermediate filaments and cellular mechanics. Cell Biol Int.

[5] Spiliotis, E.T. Spatial effects—site-specific regulation of actin and microtubule organization by septin GTPases. *J. Cell Sci.* **131** (2018). 10.1242/jcs.207555 PMC5818061 29326311

[6] Mulari MT, Zhao H, Lakkakorpi PT, Vaananen HK (2003). Osteoclast ruffled border has distinct subdomains for secretion and degraded matrix uptake. Traffic.

[7] Okumura S (2006). Coordination of microtubules and the actin cytoskeleton is important in osteoclast function, but calcitonin disrupts sealing zones without affecting microtubule networks. Bone.

[8] Turksen K, Kanehisa J, Opas M, Heersche JN, Aubin JE (1988). Adhesion patterns and cytoskeleton of rabbit osteoclasts on bone slices and glass. J Bone Miner Res.

[9] Luders J, Stearns T (2007). Microtubule-organizing centres: a re-evaluation. Nat Rev Mol Cell Biol.

[10] Bornens M, Azimzadeh J (2007). Origin and evolution of the centrosome. Adv Exp Med Biol.

[11] Cameron DA (1968). The Golgi apparatus in bone and cartilage cells. Clin Orthop Relat Res.

[12] Matthews JL, Martin JH, Race GJ, Collins EJ (1967). Giant-cell centrioles. Science.

[13] Philip, R., Fiorino, C., Harrison, R. E. Terminally differentiated osteoclasts organize centrosomes into large clusters for microtubule nucleation and bone resorption. *Mol. Biol. Cell* **33**, ar68 (2022). 10.1091/mbc.E22-03-0098 PMC9635281 35511803

[14] Tassin AM, Maro B, Bornens M (1985). Fate of microtubule-organizing centers during myogenesis in vitro. J Cell Biol.

[15] Burute M (2017). Polarity reversal by centrosome repositioning primes cell scattering during epithelial-to-mesenchymal transition. Dev Cell.

[16] Wu Q, Li B, Liu L, Sun S, Sun S (2020). Centrosome dysfunction: A link between senescence and tumor immunity. Signal Transduct Target Ther.

[17] Mulari M, Vaaraniemi J, Vaananen HK (2003). Intracellular membrane trafficking in bone resorbing osteoclasts. Microsc. Res. Tech..

[18] Lee JW (2017). The HIV co-receptor CCR5 regulates osteoclast function. Nat. Commun..

[19] Pinero J (2020). The DisGeNET knowledge platform for disease genomics: 2019 update. Nucleic Acids Res.

[20] Lu W, Gelfand VI (2017). Moonlighting motors: Kinesin, dynein, and cell polarity. Trends Cell Biol.

[21] Pu J, Guardia CM, Keren-Kaplan T, Bonifacino JS (2016). Mechanisms and functions of lysosome positioning. J Cell Sci.

[22] Del Fattore A (2008). A new heterozygous mutation (R714C) of the osteopetrosis gene, pleckstrin homolog domain containing family M (with run domain) member 1 (PLEKHM1), impairs vesicular acidification and increases TRACP secretion in osteoclasts. J Bone Miner Res.

[23] Van Wesenbeeck L (2007). Involvement of PLEKHM1 in osteoclastic vesicular transport and osteopetrosis in incisors absent rats and humans. J Clin Invest.

[24] Gundersen GG, Worman HJ (2013). Nuclear positioning. Cell.

[25] Moudjou M, Lanotte M, Bornens M (1989). The fate of the centrosome-microtubule network in monocyte-derived giant cells. J Cell Sci.

[26] Quintyne NJ, Reing JE, Hoffelder DR, Gollin SM, Saunders WS (2005). Spindle multipolarity is prevented by centrosomal clustering. Science.

[27] Taiwo BO (2015). Less bone loss with maraviroc- versus tenofovir-containing antiretroviral therapy in the AIDS clinical trials group A5303 study. Clin Infect Dis.

[28] Fux CA (2008). Tenofovir use is associated with an increase in serum alkaline phosphatase in the Swiss HIV Cohort Study. Antivir Ther.

[29] Gallant JE (2004). Efficacy and safety of tenofovir DF vs stavudine in combination therapy in antiretroviral-naive patients: A 3-year randomized trial. JAMA.

[30] Raynaud-Messina B (2018). Bone degradation machinery of osteoclasts: An HIV-1 target that contributes to bone loss. Proc Natl Acad Sci USA.

[31] Kornak U (2001). Loss of the ClC-7 chloride channel leads to osteopetrosis in mice and man. Cell.

[32] Reinholt FP (1999). Extensive clear zone and defective ruffled border formation in osteoclasts of osteopetrotic (ia/ia) rats: implications for secretory function. Exp Cell Res.

[33] Fujiwara T (2016). PLEKHM1/DEF8/RAB7 complex regulates lysosome positioning and bone homeostasis. JCI Insight.

[34] Jordens I (2001). The Rab7 effector protein RILP controls lysosomal transport by inducing the recruitment of dynein-dynactin motors. Curr Biol.

[35] Marwaha R (2017). The Rab7 effector PLEKHM1 binds Arl8b to promote cargo traffic to lysosomes. J Cell Biol.

[36] McEwan DG (2015). PLEKHM1 regulates autophagosome-lysosome fusion through HOPS complex and LC3/GABARAP proteins. Mol Cell.

[37] Murai M (2003). Peyer's patch is the essential site in initiating murine acute and lethal graft-versus-host reaction. Nat Immunol.

[38] Ito M (2005). Multi-detector row CT imaging of vertebral microstructure for evaluation of fracture risk. J Bone Miner Res.

[39] Dempster DW (2013). Standardized nomenclature, symbols, and units for bone histomorphometry: A 2012 update of the report of the ASBMR histomorphometry nomenclature committee. J Bone Miner Res.

[40] Parfitt, A. M. *et al.* Bone histomorphometry: Standardization of nomenclature, symbols, and units. Report of the ASBMR Histomorphometry Nomenclature Committee. *J Bone Miner. Res.* **2**, 595–610 (1987). 10.1002/jbmr.5650020617 3455637

[41] Dobin A (2013). STAR: Ultrafast universal RNA-seq aligner. Bioinformatics.

[42] Putri, G. H., Anders, S., Pyl, P. T., Pimanda, J. E., Zanini, F. Analysing high-throughput sequencing data in Python with HTSeq 2.0. *Bioinformatics* (2022). 10.1093/bioinformatics/btac166 PMC9113351 35561197

[43] Love MI, Huber W, Anders S (2014). Moderated estimation of fold change and dispersion for RNA-seq data with DESeq2. Genome Biol.

[44] Frankish A (2019). GENCODE reference annotation for the human and mouse genomes. Nucleic Acids Res.

[45] Pinero J (2017). DisGeNET: A comprehensive platform integrating information on human disease-associated genes and variants. Nucleic Acids Res.

