# Supplementary material for: Centrosome clustering control in osteoclasts through CCR5-mediated signaling
Source: Sci Rep. 2023 Nov 27;13:20813. doi: 10.1038/s41598-023-48140-2 (PMC10681980; doi:10.1038/s41598-023-48140-2)
Supplement: Supplementary file 1 — Supplementary Information 1. [file 41598_2023_48140_MOESM1_ESM.docx]

**Supplementary legends**

**Supplemental Figure 1. Bone phenotype from WT and *Ccr5*-deficient 10 and 25-week-old mice**

A) μCT images and the analysis of trabecular bone obtained from 10-week-old male *Ccr5*-deficient mice [n=5] and their wild type male littermates (WT) [n=5]). B) Bone volume/tissue volume ratio (BV/TV), connective density (Conn-Dens.), trabecular number (Tb.N), trabecular space (Tb.Sp) and trabecular thickness (Tb.Th) were scored by a μCT analysis. C) Calcein double-labeled images of the mineralized surface of the distal femur. Scale bars: 50 μm. D) The data included the mineral apposition rate (MAR), the bone formation rate per bone surface (BFR/BS) and the bone formation rate per bone volume (BFR/BV). Data indicate the mean ± SD. *P* value were determined by an unpaired *t*-test. E) μCT images of trabecular bone obtained from 25-week-old male C57BL/6J mice. F) BV/TV, Conn-Dens., Tb.N, Tb.Th (trabecular thickness) and Tb.Sp (trabecular separation) were scored by a μCT analysis (WT [n=4] and *Ccr5*-deficienct mice [n=5]).

**Supplemental Figure 2. *Ccr5*-deficient osteoclasts impaired bone resorption function** A) BMMs were cultured for 6 days with M-CSF (50 ng/mL) and RANKL (100 ng/mL) in WT and *Ccr5*-deficient osteoclasts. Cells were fixed and stained with phalloidin conjugated with 568 and DAPI. B, C) The number of osteoclasts per well and the number of nuclei per cell were counted. D) osteoclasts cell size was measured by ImageJ. E, F) Dentin slices were stained with hematoxylin for resorption pits formation. Intensity of pit staining was measured by ImageJ. The results are representative of at least three independent experiments.

**Supplemental Figure 3. Comparison of coupling factor genes in WT and *Ccr5*-deficient osteoclasts**

A) The transcriptional expression of coupling factor genes (*Igf1, C3, Nrp1, Fas, Tgfb3* and *Efnb1*) in WT and *Ccr5*-deficient osteoclasts was obtained from RNA sequencing data (8-week-old mice). FC means log2 fold change. B) The transcriptional expression of coupling factor genes (*Igf1, C3, Nrp1, Fas, Tgfb3, Efnb1, Sema3a, Sema4d, Efnb2, Ephb4 and Fasl*) was assessed in a co-culture system consisting of osteoblasts and osteoclasts derived from both WT and *Ccr5*-deficient mice.

**Supplemental Figure 4. Colocalization of Lamp1 and Cathepsin K in mature osteoclasts**

A) Double immunofluorescence staining of Lamp1 and Cathepsin K (shown in green and red, respectively) in WT and *Ccr5*-deficient osteoclasts cultured on plastic dishes. Scale bars; 100 μm.

**Supplemental Figure 5. Overexpression of Ccr5 in BMMs rescues impaired centrosome clustering and lysosome localization** A) Osteoclast precursors were transduced with Adv-Ccr5 overexpression for 2 days. Cells were washed and further cultured until day 6 with M-CSF and RANKL. Real-time qPCR was performed for the expression of *Ccr5*. B) Cells were subjected to immunofluorescence staining with anti-Tubulin (shown in red) and anti-Pericentrin (shown in green) with counterstaining of nuclei with DAPI (shown in blue). Scale bars: 50 μm. C) The number of dispersed centrosome per osteoclasts (arrowhead) was counted. D) Representative images of immunofluorescence staining with anti-Lamp1 (shown in green) in mature osteoclasts derived from WT and Ccr5-deficiency. Scale bars: 500 μm. E) The quantitative analysis of Lamp1 distribution across cells. The fluorescence intensity derived from Lamp1 was plotted along lines shown in E.

**Supplemental Figure 5. Colocalization of Lamp1 and Cathepsin K in mature osteoclasts**

A) Double immunofluorescence staining of Lamp1 and Cathepsin K (shown in green and red, respectively) in WT and *Ccr5*-deficient osteoclasts cultured on plastic dishes. Scale bars; 100 μm.

**Supplemental Figure 6. Schematic illustrations of cellular polarity regulated by the centrosome clustering and the establishment of MTOCs during osteoclast differentiation**

A) A centrosome in a mononuclear macrophage. The centrosome is localized adjacent to its nucleus, and provides polarized microtubule arrays in interphase cells, and mitotic spindle organization during mitosis. B) Centrosome clustering in multinucleated osteoclasts. Centrosome clustering plays a critical role in cell polarity. C) Impaired centrosome clustering in *Ccr5*-deficient osteoclasts. The loss of function of CCR5 diminishes the osteoclast function due to the impaired centrosome clustering, cytoskeletal dysplasia and consequent failure of functional cell polarity. D) The CCR5-mediated regulatory network in osteoclastogenesis.

**Supplemental Table 1. The gene ontology enrichment analysis of the molecular function of genes that were downregulated in *Ccr5*-deficent osteoclasts in comparison to WT osteoclasts**

**Supplemental Table 2. Real-time PCR amplification primers for sequencing**

**Supplemental Movie 1. Time-lapse live cell images during osteoclastogenesis of WT and *Ccr5*-deficient osteoclasts** Osteoclasts were stained with Hoechst 33342 before taking time lapse images. Time-lapse sequence images of mature osteoclasts were acquired every 10 min until apoptosis.

**Supplemental Movie 2. Lysosome trafficking in living osteoclasts was monitored by a non-staining methods using an external phase contrast system. Live images of mature osteoclasts were acquired at 10 loops per second for 50 seconds** Time-lapse images of lysosome tracking around the nucleus of WT, *Ccr5*-deficient osteoclasts, constitutive active Rho expressing *Ccr5*-deficient osteoclasts, and constitutive active Rac expressing *Ccr5*-deficient osteoclasts.
