# Supplementary material for: Centrosome clustering control in osteoclasts through CCR5-mediated signaling
Source: Sci Rep. 2023 Nov 27;13:20813. doi: 10.1038/s41598-023-48140-2 (PMC10681980; doi:10.1038/s41598-023-48140-2)
Supplement: Supplementary file 2 — Supplementary Information 2. [file 41598_2023_48140_MOESM2_ESM.pdf]

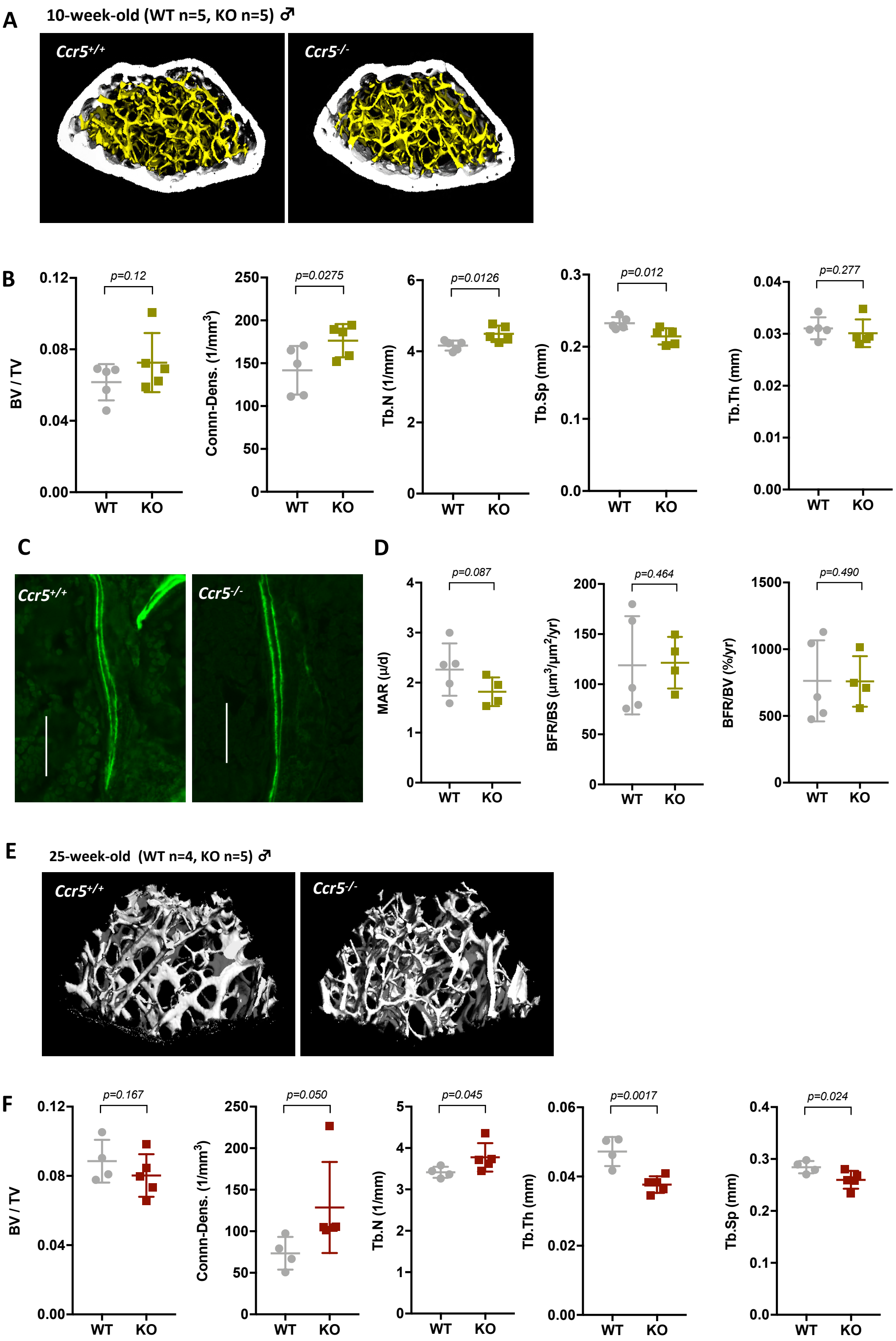
A
10-week-old (WT n=5, KO n=5) ♂
Ccr5+/+
Ccr5-/-
B
BV / TV
p=0.12
Connn-Dens. (1/mm3)
p=0.0275
Tb.N (1/mm)
p=0.0126
Tb.Sp (mm)
p=0.012
Tb.Th (mm)
p=0.277
WT
KO
C
D
MAR (μ/d)
p=0.087
BFR/BS (μm3/μm2/yr)
p=0.464
BFR/BV (%/yr)
p=0.490
E
25-week-old (WT n=4, KO n=5) ♂
F
p=0.167
p=0.050
p=0.045
p=0.0017
p=0.024

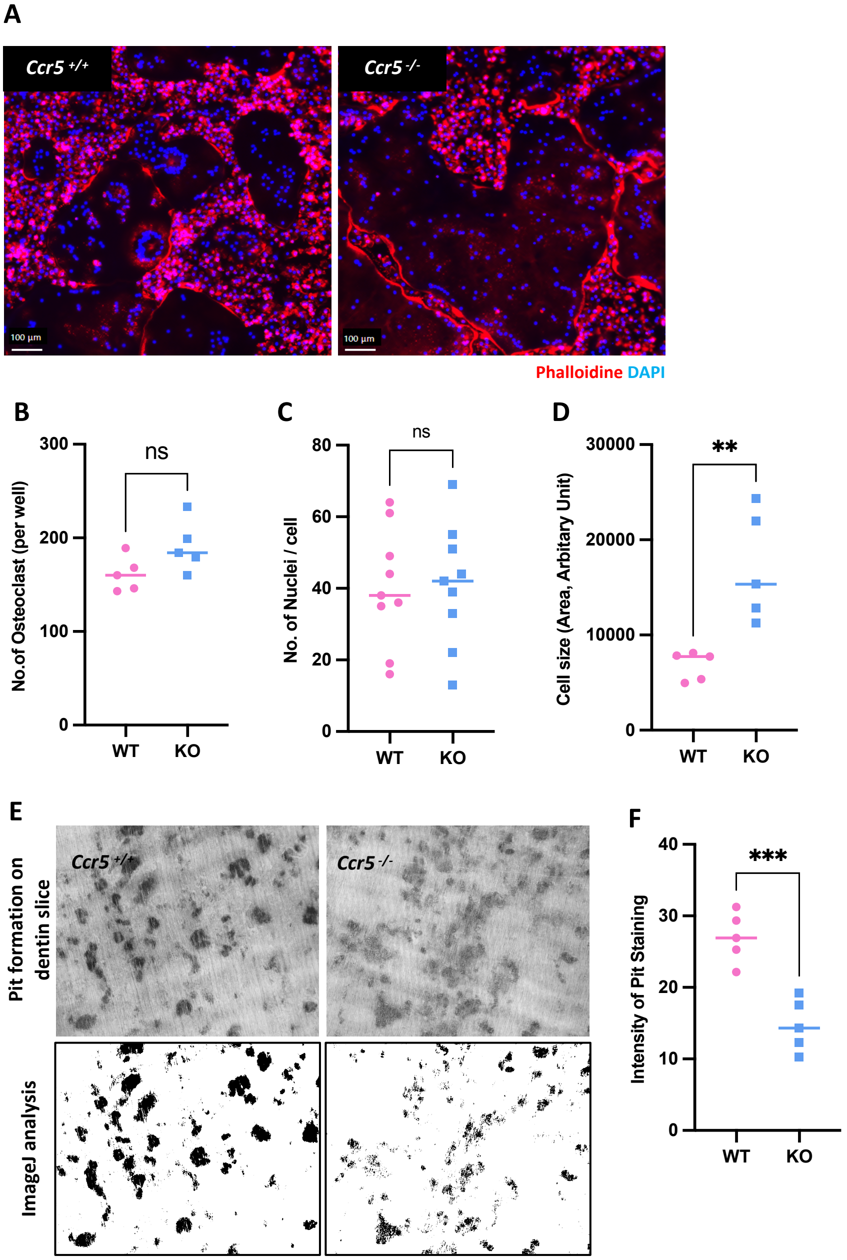
A
Ccr5 +/+
Ccr5 -/-
100 µm
100 µm
Phalloidine DAPI
B
No.of Osteoclast (per well)
ns
300
200
100
0
WT
KO
C
No. of Nuclei / cell
ns
80
60
40
20
0
WT
KO
D
Cell size (Area, Arbitary Unit)
**
30000
20000
10000
0
WT
KO
E
Pit formation on dentin slice
ImageJ analysis
Ccr5 +/+
Ccr5 -/-
F
Intensity of Pit Staining
***
40
30
20
10
0
WT
KO

A

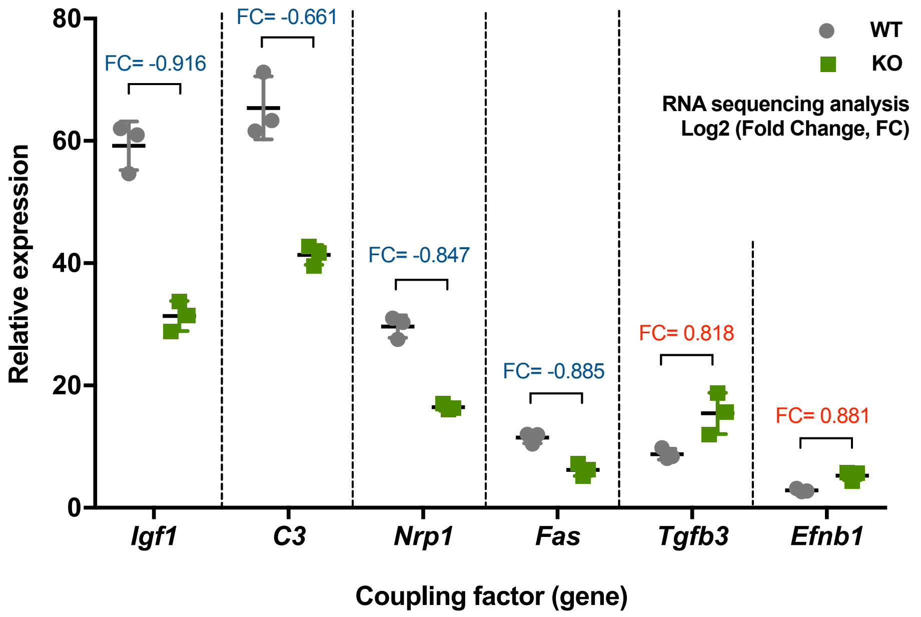
FC= -0.916
FC= -0.661
FC= -0.847
FC= -0.885
FC= 0.818
FC= 0.881
WT
KO
RNA sequencing analysis
Log2 (Fold Change, FC)
Relative expression
0
20
40
60
80
Igf1
C3
Nrp1
Fas
Tgfb3
Efnb1
Coupling factor (gene)

B

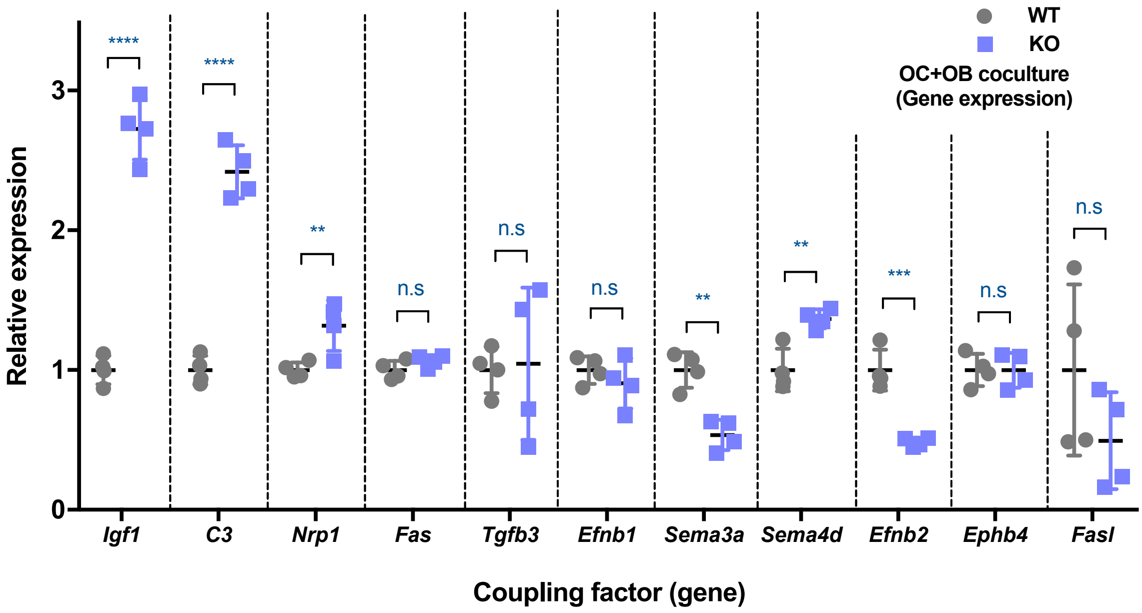
WT
KO
OC+OB coculture
(Gene expression)
****
****
**
n.s
n.s
n.s
**
**
***
n.s
n.s
Relative expression
0
1
2
3
Igf1
C3
Nrp1
Fas
Tgfb3
Efnb1
Sema3a
Sema4d
Efnb2
Ephb4
Fasl
Coupling factor (gene)

A

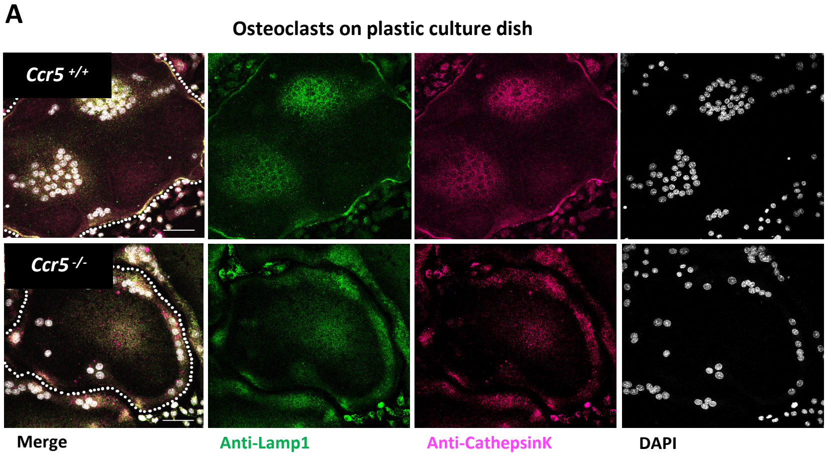

Osteoclasts on plastic culture dish
Ccr5 +/+
Ccr5 -/-
Merge
Anti-Lamp1
Anti-CathepsinK
DAPI

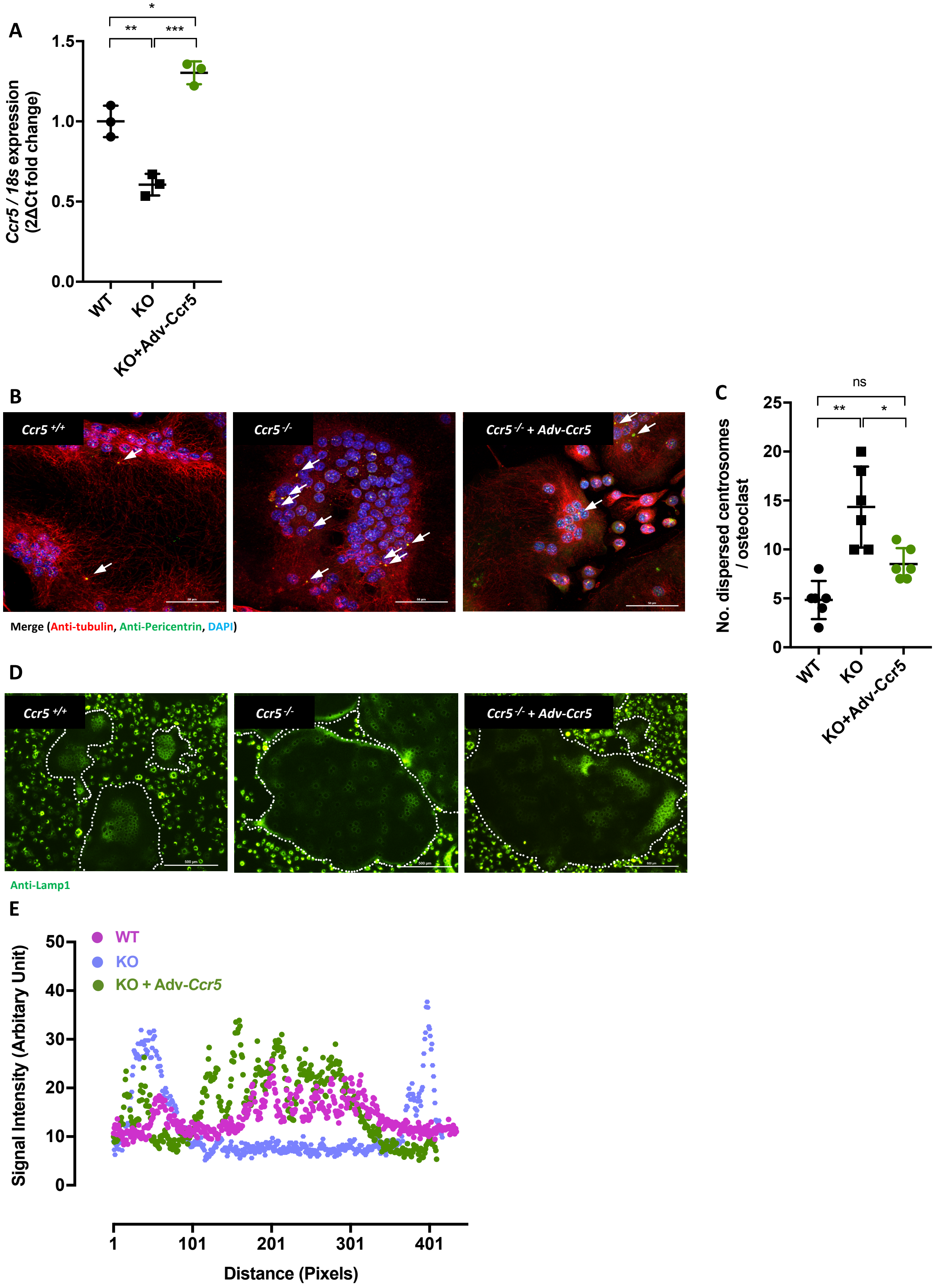
A
*
**
***
Ccr5 / 18s expression
(2ΔCt fold change)
1.5
1.0
0.5
0.0
WT
KO
KO+Adv-Ccr5
B
Ccr5 +/+
Ccr5 -/-
Ccr5 -/- + Adv-Ccr5
Merge (Anti-tubulin, Anti-Pericentrin, DAPI)
C
ns
**
*
No. dispersed centrosomes
/ osteoclast
25
20
15
10
5
0
WT
KO
KO+Adv-Ccr5
D
Ccr5 +/+
Ccr5 -/-
Ccr5 -/- + Adv-Ccr5
Anti-Lamp1
E
WT
KO
KO + Adv-Ccr5
Signal Intensity (Arbitary Unit)
50
40
30
20
10
0
1
101
201
301
401
Distance (Pixels)

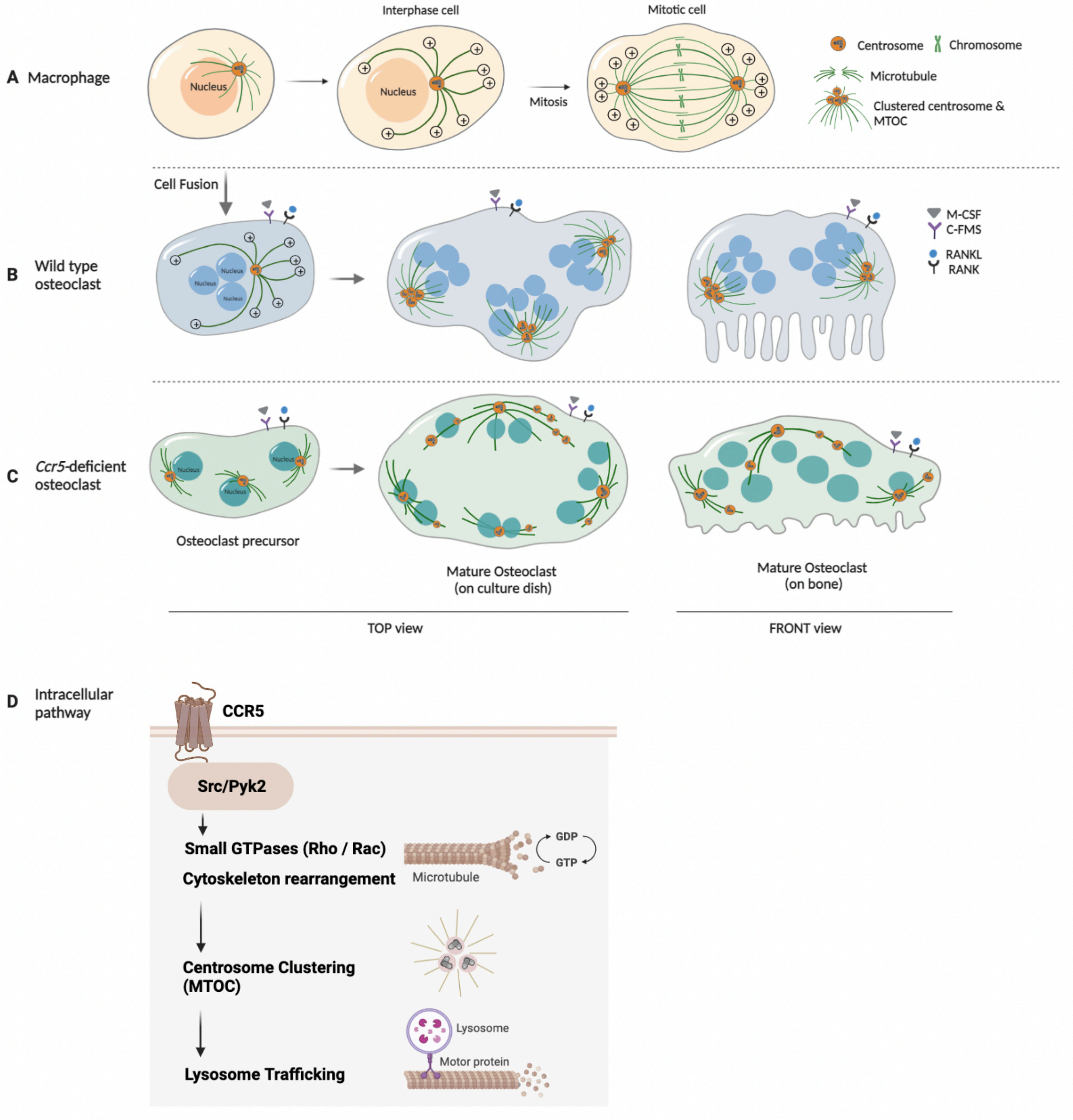
A Macrophage
Interphase cell
Mitotic cell
Nucleus
Nucleus
Mitosis
Centrosome
Chromosome
Microtubule
Clustered centrosome & MTOC
Cell Fusion
B Wild type osteoclast
M-CSF
C-FMS
RANKL
RANK
C Ccr5-deficient osteoclast
Osteoclast precursor
Mature Osteoclast (on culture dish)
Mature Osteoclast (on bone)
TOP view
FRONT view
D Intracellular pathway
CCR5
Src/Pyk2
Small GTPases (Rho / Rac)
Cytoskeleton rearrangement
Microtubule
GDP
GTP
Centrosome Clustering (MTOC)
Lysosome
Motor protein
Lysosome Trafficking

Fig.4C

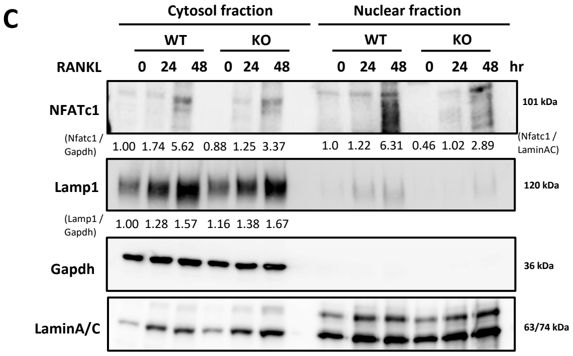

NFATc1 (101 KDa±)

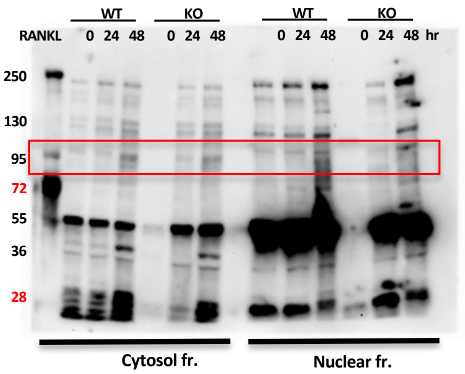

Gapdh (36 KDa)

LaminA/C (50,70 KDa)

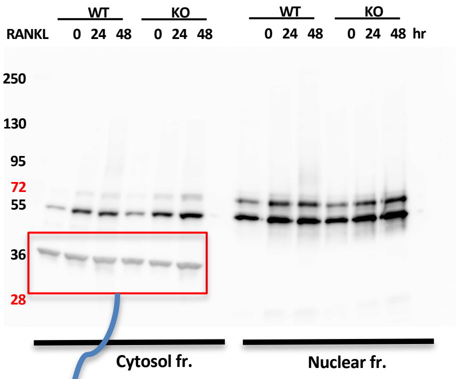

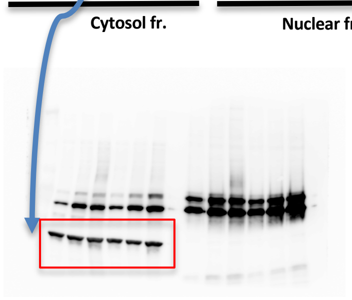

Lamp1 (120 KDa)

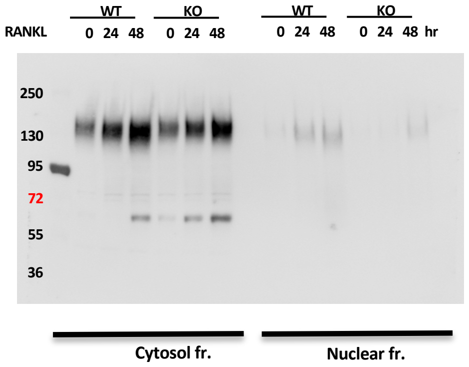

GADPH (36 KDa)

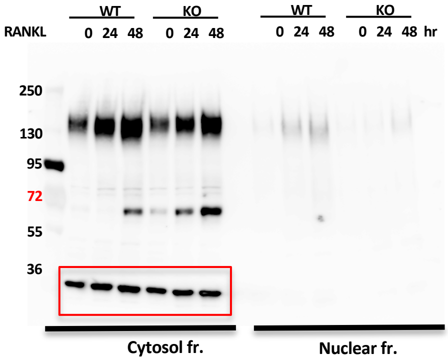

Fig.6E

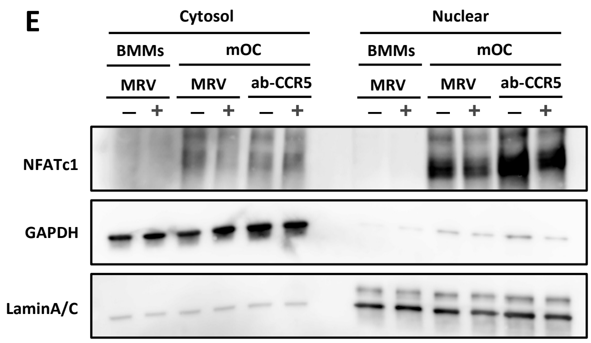
E
Cytosol
Nuclear
BMMs
mOC
BMMs
mOC
MRV
MRV
ab-CCR5
MRV
MRV
ab-CCR5
NFATc1
GAPDH
LaminA/C

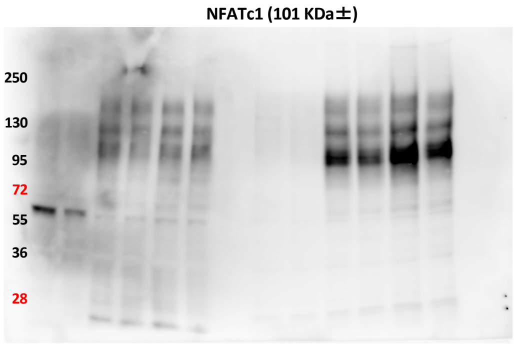
NFATc1 (101 KDa±)
250
130
95
72
55
36
28

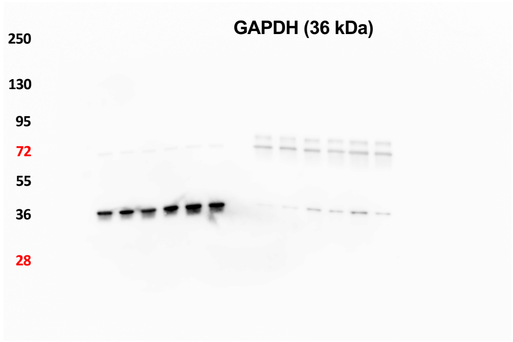
GAPDH (36 kDa)
250
130
95
72
55
36
28

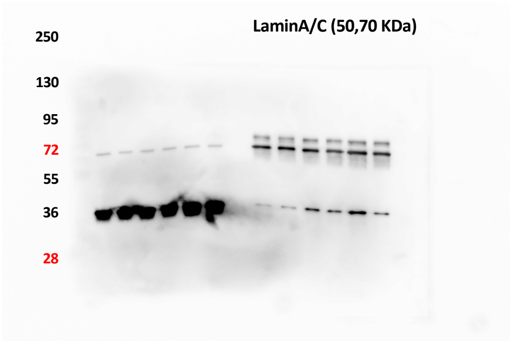
LaminA/C (50,70 KDa)
250
130
95
72
55
36
28

Term	Count	%	*P*-Value	Genes
Guanyl-nucleotide exchange factor activity	28	1.987224982	5.13E-06	DENND1C, DENND1A, ITSN1, ARHGEF10L, RASGRP4, FGD2, FGD4, AKAP13, FLCN, PREX1, CYTH4, DENND5A, FNIP1, RIC1, DENND4B, DENND4C, RAB3IL1, GAPVD1, VAV2, ALS2, MADD, ARHGEF3, ARHGEF2, DOCK2, RAPGEF6, RGL2, SOS2, ARHGEF6
GTPase activator activity	35	2.484031228	1.93E-05	ARHGAP9, TBC1D9B, FAM13B, GDI1, GDI2, ARHGAP19, ARHGAP18, ARHGAP17, ARHGEF10L, ARRB1, ARHGAP15, ARHGAP4, RASGRP3, RGS2, ADAP2, OPHN1, TBC1D14, TBC1D17, SRGAP2, TBC1D22A, GIT2, STARD8, TBC1D8, MYO9B, ARAP1, GAPVD1, ARHGAP25, ARHGAP30, ALS2, TBC1D4, RASA4, RP2, RGS10, TBC1D24, DOCK2
kinase activity	74	5.251951739	3.60E-05	SMG1, CIITA, BMPR2, CERK, ZMYND8, DGKA, AKAP8L, PIK3CD, PIK3C2A, HK3, TBK1, PIP4K2A, MAP3K8, TK2, IKBKE, PDK2, JAK1, CAMK1D, SYK, DAPK1, PRKCB, PRKCD, MINK1, DYRK1B, IRAK4, TYK2, MAPK8IP3, TGFBR2, ERN1, HCK, MOB3C, TYROBP, MAPKAPK3, MAPKAPK2, BMP2K, CMPK2, ULK2, IDNK, ULK1, SGK1, ALPK1, CSF1R, PDXK, PFKFB4, PIK3R1, STK4, DGUOK, GRK2, MKNK1, STK38, CCL3, TRPM7, IP6K1, MAPK3, MAP3K2, LYN, GIT2, CDK19, MAP3K1, CHKB, INSR, EIF2AK2, MERTK, MAPK14, CLK4, CLK1, PKIB, PIKFYVE, PINK1, FES, ETNK1, POMK, CAMK1, MAP3K12
Rab guanyl-nucleotide exchange factor activity	10	0.709723208	1.43E-04	DENND1C, RIC1, ALS2, DENND4B, DENND1A, DENND4C, MADD, DENND5A, RAB3IL1, SBF2
ATP binding	138	9.79418027	1.43E-04	EIF4A2, CERK, ATP8A1, DGKA, DQX1, TBK1, KIF5C, DHX57, DHX58, PIP4K2A, MAP3K8, KIF21B, GLUL, PDK2, DDX17, ENTPD1, DAPK1, PRKCB, PRKCD, STARD9, DYRK1B, ERN1, DHX40, NAIP2, LARS2, NAIP5, MAPKAPK3, NAIP6, TEP1, MAPKAPK2, CMPK2, ULK2, IDNK, ULK1, ALPK1, CSF1R, PFKFB4, DDX5, PFKFB3, TWF1, DDX60, STK4, DGUOK, GRK2, KIF3B, KIF3A, STK38, NLRP3, TRPM7, IP6K1, ABCA1, LYN, CDK19, CHKB, UBE2B, ABCA3, INSR, ABCA9, EIF2AK2, MYO5A, CLK4, CLK1, EHD4, FES, TRPV4, ITM2B, MAP3K12, ITM2C, ABCD4, SMG1, CIITA, ABCD2, BMPR2, CHD9, CHD6, PIK3CD, PYGL, CHD3, PIK3C2A, IFIH1, HK3, KIF13B, TK2, ABCD1, IKBKE, JAK1, OASL2, ABCC3, CAMK1D, SYK, DDX58, ABCC5, MINK1, IRAK4, TYK2, TGFBR2, HCK, KIF9, OAS2, OAS3, BMP2K, SGK1, SLFN5, NLRP1B, PDXK, RNASEL, SLFN8, SLFN2, SLFN4, NLRC5, ADCY3, OAS1A, NOD1, OAS1B, NLRC4, ADCY7, OAS1G, MKNK1, ACSS1, MAPK3, MAP3K2, MAP3K1, KCNJ10, MYO9B, ATP2B1, MAPK14, MERTK, CLCN7, P2RX7, PIKFYVE, PINK1, P2RX4, CLCN4, ETNK1, POMK, ATP13A2, CAMK1, MYO1F
GTPase activity	30	2.129169624	1.63E-04	RAB9, RAB1A, RAB5B, RAP1A, GNG2, RRAS, GNA12, RAC2, RAB29, GBP2, GM5431, 9930111J21RIK1, GBP5, TGTP2, GBP7, GBP9, MX2, MX1, RHOG, IRGM2, IRGM1, IFI47, GTPBP2, EEF1A1, RIT1, RAB14, RRAGC, RRAGD, GM4951, RHOQ
GTP binding	45	3.193754436	3.58E-04	RAB9, RAB1A, RAB5B, CIITA, RAB3D, GVIN1, RAP1A, ARL5C, RRAS, GNA12, RAC2, ANXA6, RAB29, GBP2, ARL5A, GM5431, 9930111J21RIK2, 9930111J21RIK1, RAB8B, GBP5, ARL11, TGTP2, GBP7, DAPK1, GBP9, MX2, INSR, MX1, RHOG, IRGM2, IRGM1, IFI47, GTPBP2, GM12250, EEF1A1, RIT1, RAB31, EHD4, RAB14, RRAGC, RP2, RRAGD, GM4951, UPRT, RHOQ
Rho guanyl-nucleotide exchange factor activity	15	1.064584812	3.66E-04	PLEKHG3, ITSN2, ITSN1, ARHGEF10L, FGD2, VAV2, FGD4, PREX1, AKAP13, ALS2, ARHGEF3, ARHGEF2, RGL2, SOS2, ARHGEF6
Rab GTPase binding	21	1.490418737	4.71E-04	RIC1, TBC1D9B, GDI1, DENND1A, TBC1D8, MYO5A, POT1B, WDR44, ALS2, TBC1D4, KIF3A, TMEM127, DMXL2, RAB29, TBC1D14, ULK1, DENND5A, TBC1D22A, RILP, APPL2, RAB11FIP5

Species	Gene		Sequences (5' to 3')
Mouse	*Cstk*	Forward	5' - AGCAGAACGGAGGCATTGACTC
		Reverse	5' - CCCTCTGCATTTAGCTGCCTTTG
	Plekhm1	Forwad	5' - GTGGTTTCCTCTCCTACGAGTC
		Reverse	5' - GACTCTGGAGTGTCTTGCACAG
	Rplp0	Forwad	5' - GCTTCGTGTTCACCAAGGAGGA
		Reverse	5' - GTCCTAGACCAGTGTTCTGAGC
	Igf1	Forward	5' - GTGGATGCTCTTCAGTTCGTGTG
		Reverse	5' - TCCAGTCTCCTCAGATCACAGC
	C3	Forwad	5' - CGCAACGAACAGGTGGAGATCA
		Reverse	5' - CTGGAAGTAGCGATTCTTGGCG
	Nrp1	Forwad	5' - CGGAGGAATGTTCTGTCGCTATG
		Reverse	5' - GGATAGAACGCCTGAAGAGGAG
	Fas	Forward	5' - CTGCGATTCTCCTGGCTGTGAA
		Reverse	5' - CAACAACCATAGGCGATTTCTGG
	Tgfb3	Forwad	5' - AAGCAGCGCTACATAGGTGGCA
		Reverse	5' - GGCTGAAAGGTGTGACATGGAC
	Efnb1	Forwad	5' - TGTGTCGCACCCGCACTATGAA
		Reverse	5' - GACAGTGTTGTCTGACTCCTTGC
	Fasl	Forward	5' - GAAGGAACTGGCAGAACTCCGT
		Reverse	5' - GCCACACTCCTCGGCTCTTTTT
	Sema3a	Forwad	5' - GACATCTATGGCAAAGCCTGTGC
		Reverse	5' - GTGAGTCAGTGGGTCTCCATTC
	Sema4d	Forwad	5' - CCAGATAGTGGTAGACAGGACC
		Reverse	5' - GTCTCCTCGATGACATGCACCT
	Efnb2	Forwad	5' - CCAACAAGACGTCCAGAGCTAG
		Reverse	5' - CCACTTCGGAACCCAGGAGATT
	Eohb4	Forwad	5' - GTGCTGGACTACGAGGTCAAGT
		Reverse	5' - TACCTGGACCAGATAGCTGGCT
Human	*CCR5*	Forwad	5' - TCCTGCCTCCGCTCTACT
		Reverse	5' - GAACTTCTCCCCGACAAA
	TRAP	Forwad	5' - GATCCTGGGTGCAGACTTCA
		Reverse	5' - GCGCTTGGAGATCTTAGAGT
	CD4	Forwad	5' - CCTCCTGCTTTTCATTGGGCTAG
		Reverse	5' - TGAGGACACTGGCAGGTCTTC T
	PLEKHM1	Forwad	5' - CTGAAGCTGGTAGTTTCCTCACC
		Reverse	5' - GCTATTGGGTGGTCTGAAGCAC
	RPLP0	Forwad	5' - TGGTCATCCAGCAGGTGTTCGA
		Reverse	5' - ACAGACACTGGCAACATTGCGG
